# Multivariate Risk Analysis of RAS, BRAF and EGFR Mutations Allelic Frequency and Coexistence as Colorectal Cancer Predictive Biomarkers

**DOI:** 10.3390/cancers14112792

**Published:** 2022-06-04

**Authors:** Adriana Ionescu, Liviu Bilteanu, Ovidiu Ionut Geicu, Florin Iordache, Loredana Stanca, Aurelia Magdalena Pisoschi, Adrian Miron, Andreea Iren Serban, Valentin Calu

**Affiliations:** 1 Department of Biochemistry and Molecular Biology, Faculty of Biology, University of Bucharest, 91-95 Blvd., Splaiul Independentei, 050095 Bucharest, Romania; a.ionescu20@s.bio.unibuc.ro; 2 Department of Preclinical Sciences, Faculty of Veterinary Medicine, University of Agronomic Sciences and Veterinary Medicine of Bucharest, 105 Blvd., Splaiul Independentei, 050097 Bucharest, Romania; liviu.bilteanu@fmvb.usamv.ro (L.B.); ovidiu-ionut.geicu@fmvb.usamv.ro (O.I.G.); florin.iordache@fmvb.usamv.ro (F.I.); loredana.stanca@fmvb.usamv.ro (L.S.); aurelia-magdalena.pisoschi@fmvb.usamv.ro (A.M.P.); 3 Taxon Solutions SRL, 7 Semilunei Street, 020797 Bucharest, Romania; 4 Department of General Surgery, University of Medicine and Pharmacy “Carol Davila” Bucharest, 8 Blvd., Eroii Sanitari, 050474 Bucharest, Romania; adrian.miron@umfcd.ro (A.M.); valentin.calu@umfcd.ro (V.C.); 5 Department of Surgery, “Elias” Emergency University Hospital, 17 Marasti Blvd., 01146 Bucharest, Romania

**Keywords:** colorectal cancer, ddPCR, KRAS, NRAS, BRAF, EGFR

## Abstract

**Simple Summary:**

The colorectal cancer (CRC) stage and evolution should be described by biomarker profiles. In 60 CRC cases, KRAS, NRAS, BRAF, and EGFR mutations were analyzed by droplet digital PCR (ddPCR). KRAS G12/G13 mutation was present in all patients with variable allelic frequencies. KRAS Q61 mutation was correlated with tumor invasion beyond the subserosa and poor differentiation, both associated with worst prognosis. Tumors with NRAS and BRAF mutations were prevalently localized on the right segment colon. The discovery of the KRAS Q61 role in tumor phenotypes provides the foundation for new therapeutic strategies and perspectives on molecular subtypes classification of CRC.

**Abstract:**

Background: Biomarker profiles should represent a coherent description of the colorectal cancer (CRC) stage and its predicted evolution. Methods: Using droplet digital PCR, we detected the allelic frequencies (AF) of KRAS, NRAS, BRAF, and EGFR mutations from 60 tumors. We employed a pair-wise association approach to estimate the risk involving AF mutations as outcome variables for clinical data and as predicting variables for tumor-staging. We evaluated correlations between mutations of AFs and also between the mutations and histopathology features (tumor staging, inflammation, differentiation, and invasiveness). Results: KRAS G12/G13 mutations were present in all patients. KRAS Q61 was significantly associated with poor differentiation, high desmoplastic reaction, invasiveness (ypT4), and metastasis (ypM1). NRAS and BRAF were associated with the right-side localization of tumors. Diabetic patients had a higher risk to exhibit NRAS G12/G13 mutations. BRAF and NRAS G12/G13 mutations co-existed in tumors with invasiveness limited to the submucosa. Conclusions: The associations we found and the mutational AF we reported may help to understand disease processes and may be considered as potential CCR biomarker candidates. In addition, we propose representative mutation panels associated with specific clinical and histopathological features of CRC, as a unique opportunity to refine the degree of personalization of CRC treatment.

## 1. Introduction

Evidence-based medicine has paved the way for a paradigm in which biomarker profiles are used to create a coherent description of the health status within a personalized medicine [1], with predictive results. As a result, prevention is beginning to play an increasingly important role.

Many metabolic, immunologic, or therapeutic factors regulate the tumor progression by influencing the development of a microenvironment containing cells with heterogenic genetic phenotypes and behaviors [2]. Cellular heterogeneity refers to distinct populations of cells in the same tumor microenvironment, displaying various phenotypical features. On the other hand, genetic heterogeneity is determined in many cases by genomic instability, leading to a high mutation frequency in several genes. This heterogeneity is frequent in cancer patients and is crucial for acquired resistance to therapy, a common cause of relapse [3]. Many patients harbor mutations in different genes and in different exons within the same gene [4]. Thereby, the intratumor heterogeneity raises significant challenges in using molecular prognostic markers to select the patients that might benefit from specific therapies [3]. The accurate characterization of the genomic landscape of colorectal cancers (CRC) could identify the distinctive metastasis signature and increase the life expectancy of CRC patients [5].

Colorectal cancers may develop through three main mechanisms: chromosomal instability (CIN), CpG islands methylation phenotype (CIMP), and microsatellite instability (MSI). According to the Consensus molecular subtypes classification, there are four CRC subtypes and one mixed subtype without any clear designation [6,7]. CMS subtype 1 tumors display a higher percentage of MSI (74%) and CIMP (67%); high BRAF (42%) and lower KRAS (23%) gene mutation status; low somatic copy number alteration (SCNA), immune infiltration, and activation; and worse prognosis, representing 14% of total CRC [6,7]. CMS subtype 2 contributes to 37% of all tumor subtypes. These tumors have high SCNA, are microsatellite stable (MSS); have WNT- and Myc-activated pathways and elevated gene expression of EGFR; and carry mutated p53 gene and KRAS mutations (28%). CMS subtype 3 represents 13% of all tumors, with epithelial characteristics and moderately activated WNT and Myc signaling pathways along with the overexpression of IGBP3. About 68% of tumors harbor mutations in the KRAS gene and only 7% in the BRAF gene, with moderate or low MSI and intermediate CIMP status. CMS subtype 4 exhibits upregulation of genes involved in EMT transition, intense stromal infiltration, and low KRAS (28%) and BRAF (7%) mutation frequency [6,7].

The main predictive biomarkers for the response to anti-EGFR monoclonal antibodies-targeted therapy in metastatic CRC are mutations in the KRAS, NRAS, and BRAF genes [8,9] that explain the low percentage of CRC patients (10–20%) responsive to anti-EGFR monoclonal antibodies single treatment [4]. Kristen-RAS (KRAS) and neuroblastoma-RAS (NRAS) belong to the G protein type called the RAS superfamily. In normal cells, RAS protein is inactive (linked to GDP) and can become activated (linked to GTP) by many cellular receptors (tyrosine kinase receptors, G protein-coupled receptors, and integrin receptors). One such activator of RAS is EGFR, which initiates a signaling cascade [10,11]. As a result, CRC development- and progression-related signaling pathways such as MAPK, PI3K-AKT/mTOR, or Wnt/β catenin are activated [12]. Half of all CRCs harbor KRAS and NRAS activating mutations, often located in codons 12, 13, 59, and 61, that affect the metabolism of cancer cells and drive resistance to commonly used drugs [13,14,15]. Mutations in the BRAF gene are localized in exon 15 and are represented by valine amino acid substitution (V600E, V600K, and V600R). They have been described as a prognostic marker or a predictive factor for resistance to anti-EGFR monoclonal antibodies [4]. A recent study showed that proximal colon tumor localization exhibited a significant correlation with mutations in KRAS and BRAF [8]. Another study found an association between mucinous adenocarcinoma and KRAS mutations, but not with NRAS or BRAF mutations [16]. Moreover, cohort studies that sought out to link demographic data and KRAS mutational status reported contradictory results [17,18].

The effective detection of CRC-related mutations requires snap-frozen tumor samples, which are rarely available. In contrast, the formalin fixation significantly damages DNA integrity in the widely available paraffin-embedded tissue samples. Moreover, the wild-type tumor cells and non-tumor cells may outnumber mutant tumor cells, restricting the diagnosis of CRC gene-related mutations in clinical settings [10]. The ddPCR was shown to be more reliable, detecting the EGFR mutation presence at levels up to 10-fold lower in comparison to qPCR [19]. Other mutation detection technologies such as pyrosequencing and Sanger sequencing were shown to be less sensitive than ddPCR, which was used to identify 12.5% and 23% false negative results respectively [20]. In a recent meta-analysis study, ddPCR was shown to be more accurate than next generation sequencing (NGS) [21].

Therefore, we used fresh-frozen tumor samples and the highly precise Droplet Digital PCR (ddPCR) technique to overcome such limitations.

We performed a robust and detailed multivariable analysis that involves demographic and clinico-pathological data; histopathological features; and allelic frequencies of KRAS, NRAS, BRAF, and EGFR genetic mutations, which we used to generate a cluster model of CRC patients as a starting point for the improvement of targeted therapeutic strategies.

## 2. Materials and Methods

### 2.1. Patients

This study was performed per the Declaration of Helsinki 1975, amended in 2013. All protocols and methods were reviewed and approved by the Medical Ethics Committee of Elias University Emergency Hospital of Bucharest, Romania (no: 5939/2019). Before being included in the study, a written informed consent was signed by all participant patients.

Our patient cohort included 60 patients whom underwent surgery to remove colorectal tumors at Elias University Emergency Hospital between September 2019 and November 2021. No patient received preoperative chemo- or radiotherapy.

Patient clinical and demographic characteristics are reported in Table S1. The average and median ages were 67.16 and 68.5 years, 43.3% patients of in the analyzed cohort were females and 56.7% were males, 70.7% of cases (41) involved left colon neoplastic lesions, most of these (52) being staged ypT3-T4, with almost half of them exhibiting a positive node N0-N1. Both metastatic and non-metastatic staged patients were included in the study. The body mass index (BMI) of patients has been classified according to the World Health Organization’s classification of obesity [22]. Accordingly, most of the patients, 71.6% (41), were overweight (BMI ≥ 25, mean 26.74 ± 3.97). Although obesity and CRC incidence rates are increasing, the relationship between BMI, cancer recurrence, and patient survival is still unknown [23]. Furthermore, over 50% of our patients were also diagnosed with diabetes. Thus, as shown previously [24], the connection between CRC-related mutation and diabetes merits to be thoroughly explored.

The CRC epidemiologic characteristics in Romania were well represented by the clinical, demographical, and histopathological variables. This cohort may be representative for other European regions, given the general ageing population trend in Europe [4,16,17].

The excised tumor samples were assessed by hematoxylin-eosin (HE) staining to evaluate the histopathologic modifications, establish the staging, and evaluate the immune infiltrate. Additionally, resected tissue samples were analyzed by ddPCR for KRAS G12/G13, KRAS Q61, NRAS G12/G13, NRAS Q61, and BRAFV600 gene mutations, and EGFR exon 19 deletions.

### 2.2. Genomic DNA (gDNA) Isolation from Fresh Frozen CRC Tissue Samples

Tumor tissue and adjacent normal mucosa from each patient were excised and immediately frozen at − 80 °C until analysis. The DNA samples were extracted from frozen tumors tissues, in order to prevent artefacts such as C > T and DNA alteration that occur when DNA is extracted from fixed and paraffin embedded samples. According to the manufacturer’s protocol, genomic DNA (gDNA) was isolated from 10 mg of each tumor’s frozen tissue sample in triplicate using GeneJET Genomic DNA Purification Kit (Thermo Fisher Scientific, Vilnius, Lithuania, EU). The median concentration of the gDNA was 143.3 ng/µL, and its range was 32.1–1558.3 ng/µL.

### 2.3. Restriction Digest of the Isolated gDNA Sample Prior to Droplet Generation

According to the manufacturer’s recommendations, the gDNA samples analyzed for KRAS G12/G13, KRAS Q61, NRAS G12/13, and BRAF V600 NRAS Q61 mutations and EGFR Exon 19 deletions were digested 2–3 h at 37 °C in the presence of Nde I, Hind III, Hae III, and EcoR I restriction enzymes (20 U/μg gDNA), respectively.

### 2.4. CRC Tumors Tissue KRAS G12/G13, KRAS Q61, NRAS G12/G13, NRAS Q61, EGFR Exon 19 Deletion and BRAF V600 Mutations Analysis via Droplet Digital PCR

The Droplet Digital PCR (ddPCR) was performed using the QX200 Droplet Digital PCR System (Bio-Rad, Hercules, CA, USA) and the ddPCR KRAS G12/G13 Screening Multiplex Kit, ddPCR KRAS Q61 Screening Kit, ddPCR NRAS G12/G13 Screening Kit, ddPCR NRAS Q61 Screening Kit, ddPCR BRAF V600 Screening Kit, and ddPCR EGFR Exon 19 deletions Screening Kit (Bio-Rad, Hercules, CA, USA).

All restricted gDNA samples were analyzed in triplicate, and each ddPCR reaction mixture contained the 1X screening kit assay reagent, which contains a primers-probes optimized mix; wild-type probes were labelled with HEX dye, and the mutant probes were labelled with FAM dye, 1X ddPCR Supermix for probes, and 6 µL of gDNA template (8 ng/μL), adjusted to a final volume of 20 µL with DEPC-treated water. The ddPCR reaction mixture samples were mixed with 70 µL of droplet generator oil for probes (Bio-Rad, Hercules, CA, USA) and partitioned into up to 20,000 droplets using QX200 droplet generator (Bio-Rad, Hercules, CA, USA). Emulsified samples were transferred on 96-well plates, and PCR was performed on a C1000 Touch Thermal Cycler (Bio-Rad, Hercules, CA, USA). The thermal cycling conditions were: 95 ℃ for 10 min, and 40 cycles of 94 °C for 30 s, 55 °C for 1 min, and 98 °C for 10 min with a ramp rate of 2 °C/s, according to the manufacturer’s recommendations. After that, the fluorescence of the samples was read using the QX200 droplet reader (Bio-Rad, Hercules, CA, USA), selecting FAM and HEX channels. Every ddPCR run included a negative template control (Wild Type Reference Standard, for each analyzed mutation, used at a concentration similar to the samples, Horizon Discovery, Cambridge, UK) and positive template control (Quantitative Multiplex Reference Standard gDNA, covering the BRAF, KIT, EGFR, KRAS, NRAS, and PIK3CA genes with allelic frequencies between 1–24.5%, 50 ng/μL used at a concentration similar to the samples analyzed, Horizon Discovery, Cambridge, UK) for computing fluorescence thresholds. The data were analyzed with the QuantaSoft Analysis Pro Software v.1.2.10.0 (Bio-Rad, Hercules, CA, USA), providing absolute quantification of target DNA (target copies/µL of reaction). Wells with less than 10,000 accepted droplets were excluded from the analyses. The mutation allele frequency (*AF*‰, the number of mutant haploid genomes in a total of 1000 haploid genomes) was calculated using the mutant allele concentration in copies/µL (Mut) and the wild-type allele concentration in copies/µL (WT) using the equation:

$$AF\left(‰\right)=\frac{Mut}{Mut+Wt}\times 10^{3}$$

The *AF*‰ descriptive statistics for all studied mutations are presented in Table S2.

Considering that the copy-number of a fresh tumor sample varies with the amount of background wild-type gDNA by comparing with FFPE tumor tissues that have >85% tumor cells, at least two positive droplets for each investigated mutation in a triplicate had to be present for calling a sample positive for a given mutation [25,26,27]. The threshold was manually set based on positive control samples for each channel, and the threshold for positivity was ≥ 0.1 mutant copies for 10^3^ haploid genomes for all assays. Based on the “Rule of Three (3 positive mutant copies out of 3000 copies)” [28,29], the mutant positive samples were divided into two categories, one that has *AF*‰ ≥ 1 and the second with *AF*‰ ≥ 0.1 ranging from 0.1–0.99‰.

Due to its ability to accurately quantify mutations with low allelic levels, the ddPCR technique has promising potential to be integrated into medical practice as a sensitive prognostic tool.

### 2.5. Statistics Analyses

#### 2.5.1. Classes of Variables Used in This Study

For this study, we classified statistical variables into the following categories: (I) clinical variables (e.g., weight; BMI; risk comorbidities—diabetes, hypertension; risk behaviors such as smoking); (II) pathology variables: (a) macroscopic (e.g., tumor location, tumor volume, number of examined lymph nodes, invasion of other organs, etc.) and (b) microscopic (e.g., tumor differentiation grade, histopathological phenotype, tumor invasion, histopathological staging); and (III) genetic variables (e.g., number of mutant copies detected per 1000 copies of haploid genomes; etc.).

#### 2.5.2. Data Transformations

Among the variables mentioned above, there were some variables defined over untransformed values, derived from “raw data” such as age, gender, weight, etc. or data such as histopathological phenotype, tumor differentiation grade, and number of mutant copies reported per 1000 copies of haploid genomes. The values of the latter variables were obtained after preliminary processing performed according to the experimental protocols. Another category of variables was those defined by data transformation through different procedures such as the logarithmic transformation of numerical variables or the transformation of some quantitative variables into qualitative variables by defining categories using characteristic position parameters (mean; median; percentiles 25%, 33%, 50%, 67%, and 75%; or limit values observed by visual inspection of numerical data that were grouped into particular categories).

#### 2.5.3. Risk Estimation

The contingency of the ordered qualitative variables was described by calculating the odds ratio (OR) and relative risk (RR), considering as significant correlations for which at least one of these two parameters had values over 1.3. The formulas for these parameters are presented in the first section of the Supplementary Materials, Equations (S3)–(S7).

#### 2.5.4. Mutations Coexistence

Starting from the hypothesis that the studied mutations can influence each other, we estimated the risk that these mutations exist or are absent simultaneously, two by two. In addition, we calculated the risk that the presence of one mutation would expressly exclude the presence of another mutation. The two ways in which we analyzed the coexistence of mutations was defined by Equations (S1) and (S2) in the first section of Supplementary Materials. Table S3 shows the risk estimation and interpretation when applying the model defined by the Equation (S2), and Figure S1 shows the results corresponding to this model.

#### 2.5.5. Predictor and Outcome Variables

We considered all possible correlations between (a) clinical and genetic variables as independent variables on the one hand and (b) histopathological variables and genetic variables as dependent variables, on the other hand. All the values of the histopathological variables were listed in Table S4, and the complete risk estimation of their association with mutations studied was detailed in Table S5.

#### 2.5.6. Statistical Analysis Steps

Statistical analysis using the IBM SPSS Statistics 26 statistical analysis package was performed: normality checks, log transformations for data normalization, comparisons, and correlations. Our objective was to assess the statistically significant correlations between clinical, demographic, and histopathology data and all mutations.

## 3. Results

### 3.1. Mutations’ Prevalence and the Coexistence of Mutations

All CRC tumors had at least one mutation with an *AF*‰ ≥ 0.1, and 96.7% of them presented at least one mutation with *AF*‰ ≥ 1 in any of the four genes investigated; the KRAS G12/G13 was the most prevalent mutation detected in our cohort, followed by NRAS G12/G13 (25%, *AF*‰ ≥ 1) and KRAS Q61 (21.7%, *AF*‰ ≥ 1) (Table 1). In addition, we identified BRAF mutations in 25% of tumors (*AF*‰ ≥ 0.1), but only 11.7% of them had *AF*‰ ≥ 1. The EGFR exon 19 deletions were present in only 3 tumor samples in our cohort of 60 patients. Table S2 provides detailed descriptive statistics of *AF*‰ mutations.

When considering *AF*‰ ≥ 1, we identified 28 tumors with two types of mutations and only 10 tumors with three different mutations (Table 2). Moreover, 73.3% of tumors presented both KRAS G12/G13 and KRAS Q61 mutations (*AF*‰ ≥ 0.1), and only 33.3% had a coexistence for NRAS G12/G13 and NRAS Q61 mutations (*AF*‰ ≥ 0.1) (Table 3). Interestingly, at *AF*‰ ≥ 1, only 13 tumors (21.66%) carried mutations in exon 2 and exon 3 of the KRAS gene. In the case of NRAS, only two tumors (3.33%) presented both NRAS G12/G13 and NRAS Q61. The KRAS G12/G13 and BRAFV600 mutations co-occurred in only eight tumors (13.3%). In addition, we found 19 tumors (31.66%) with simultaneous mutations in two genes and five tumors with concomitant mutations in KRAS, NRAS, and BRAF genes, and one tumor with concurrent mutations in KRAS, NRAS, and EGFR genes with *AF*‰ ≥ 1.

To report the mutations coexistence, we formulated risk estimation parameters OR, RR1, RR2, and RR3, as defined by the Equations (S3)–(S7) in Supplementary Materials and interpreted according to Table S2.

**Figure 1:**
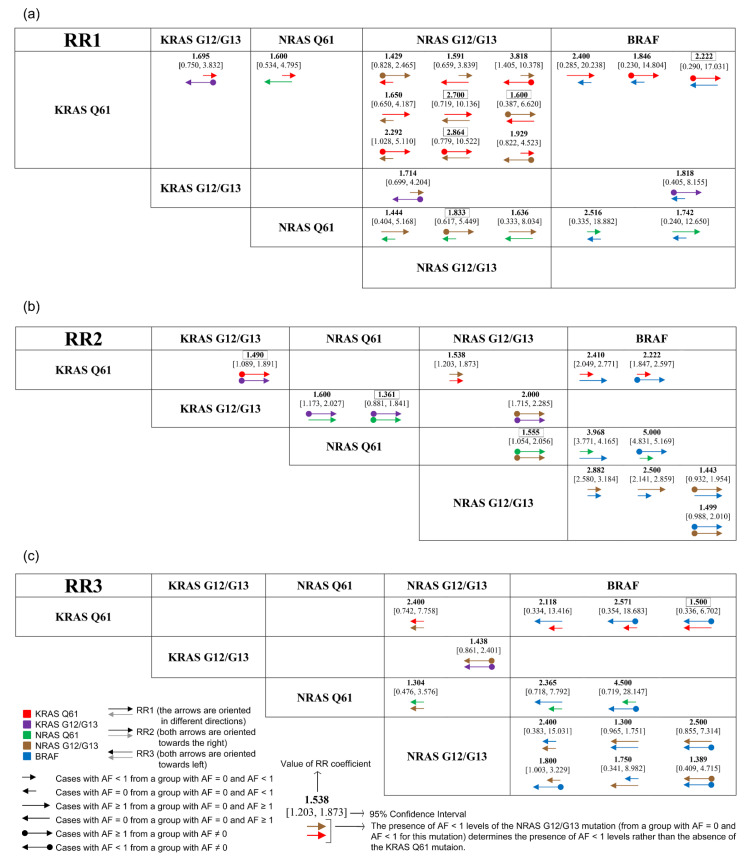
Risk analysis via RR1 (**a**), RR2 (**b**), and RR3 (**c**) of all possible two-by-two associations between the studied mutations. The section “coexistence of mutations and their associations with clinical data” in the Supplementary Materials file contains the equations defining RR1, RR2, and RR3. Table S3 contains the interpretation of these risk estimators. We selected the statistically significant values from the data presented in Supplementary Materials Figure S1.

First, we analyzed the coexistence of mutations by calculating the OR, RR1, RR2, and RR3 in terms of the presence (*AF*‰ ≥ 0.1) or absence (*AF*‰ = 0) of the mutations. Secondly, we explored the possibility of associations between different levels of the mutations. Thus, for each of the mutations, we segregated the group of patients into subgroups defined by *AF*‰, as follows: group A (cases with *AF*‰ = 0 and cases with 0.1 ≤ *AF*‰ < 1, hereinafter referred to *AF*‰ < 1), group B (cases with *AF*‰ = 0 and cases *AF*‰ ≥ 1), and group C (cases with *AF*‰ ≠ 0, with *AF*‰ < 1 and *AF*‰ ≥ 1). The KRAS G12/G13 mutations were ubiquitous in our cohort and for this mutation we evaluated only the cases included in group C. However, for the KRAS Q61, NRAS Q61, NRAS G12/G13, and BRAF mutations, we distinguished six situations relative to the A, B, and C groups: patients with *AF*‰ < 1 from group A, patients with *AF*‰ = 0 from group A, patients with *AF*‰ ≥ 1 included in group B, patients with *AF*‰ = 0 from group B, patients with *AF*‰ < 1 included in group C, and respectively patients with *AF*‰ ≥ 1 from group C. Consequently, 26 categories defined by the type of mutation and the level of the mutation (*AF*‰) were obtained. After this stratification of cases, we estimated all possible associations between *AF*‰ < 1, *AF*‰ ≥ 1, and *AF*‰ = 0 for any combination of two mutations. In other words, the number of possible associations was 325 (combinations of 26 categories taken in pairs), presented in Figure S1. Statistically significant associations are shown in Figure 1.

The absence of KRAS Q61 mutation (*AF*‰ ≥ 0.1) favored the presence of NRAS Q61 mutation with RR1 = 1.375 (95% CI = [0.786, 2.406]). However, the analysis according to *AF*‰ levels revealed that the absence of *AF*‰ ≥ 1 levels of NRAS Q61 favored the presence of KRAS Q61 mutation with *AF*‰ < 1 (RR1 = 1.600, Figure 1a). KRAS Q61 and NRAS G12/G13 mutations, with *AF*‰ < 1, coexisted (RR1 = 3.818, RR2 = 1.538, and RR3 = 2.400, Figure 1), however, when the *AF*‰ ≥ 1, the mutations were mutually exclusive as shown by the RR1 values illustrated in Figure 1a. The BRAF mutation with *AF*‰ ≥ 1 favored only the presence of KRAS Q61 mutation with *AF*‰ <1 (RR2 = 2.410, Figure 1b). The absence of the BRAF mutation (*AF*‰ < 1) was favorable to the KRAS Q61 mutation with *AF*‰ ≥1 (RR1 = 2.400, Figure 1a). The presence of the NRAS G12/G13 mutation, regardless of its AF (*AF*‰ ≥ 0.1), favored the presence of the BRAF mutation OR = 6.323 (IC 95% = [0.755, 52.920]) and RR2 = 4.673. In addition, BRAF mutation with *AF*‰ <1 can also coexist with NRAS G12/G13 mutation with both *AF*‰ ≥ 1 or *AF*‰ < 1, but the association with low *AF*‰ NRAS G12/G13 was much stronger (RR2 = 2.882 and RR3 = 2.400, Figure 1). NRAS Q61 and BRAF mutations with AF in the same value range were not found to coexist (e.g., for levels *AF*‰ < 1, RR1 = 2.516). The NRAS Q61 mutation with *AF*‰ < 1 co-occurred with BRAF mutation in tumors that express this mutation with an *AF*‰ ≥1 (RR2 = 5.000), while the *AF*‰ <1 levels of BRAF mutation were associated with the absence of the NRAS Q61 mutation with *AF*‰ < 1 (RR3 = 4.500).

The BRAF mutation with an *AF*‰ ≥ 1 was found to be associated with NRAS Q61 mutation, with an *AF*‰ < 1 (RR2 = 3.968), and in the absence of these, NRAS Q61 mutations frequencies favored the absence of BRAF mutation with an *AF*‰ ≥ 1 (RR3 = 2.365) (Figure 1). Overall, at *AF*‰ ≥ 0.1, the absence of any NRAS type mutations was correlated with the absence of BRAF mutation RR3 = 2.333 (95% CI = [0.312, 17.452]), while their concomitant occurrence was characterized by OR = 2.579 (95% CI = [0.291, 22.884]) and RR2 = 2.155. The KRAS G12/G13 mutations with *AF*‰ ≥ 1 were associated with AF levels in the same range of KRAS Q61 (RR2 = 1.490), NRAS Q61 (RR2 = 1.600), and NRAS G12/13 (RR2 = 2.000) mutations mainly when AF ≠ 0 (Figure 1b). However, the KRAS G12/G13 mutations with *AF*‰ < 1 were associated with AF in the same range only for KRAS Q61 (RR1 = 1.695) and NRAS G12/G13 (RR1 = 1.714 and RR3 = 1.438) mutations. In addition, the absence of *AF*‰ < 1 of BRAF mutations favored the presence of KRAS G12/G13 mutations with *AF*‰ ≥ 1 (RR1 = 1.818, Figure 1a).

Our results challenge the exclusion of the BRAF mutation or any other mutations by the presence of KRAS G12/G13 mutations [17,30]. Moreover, we propose that more consideration should be given to the AF of analyzed genes in the context of mutation co-existence studies. The coexistence of mutations with different AF levels also opens up perspectives for further refining the risk stratification in the target population and personalized therapeutic decisions in patients with AF levels of mutations indicating the disease progression.

### 3.2. Association between Clinical Data and Mutational Status

**Figure 2:**
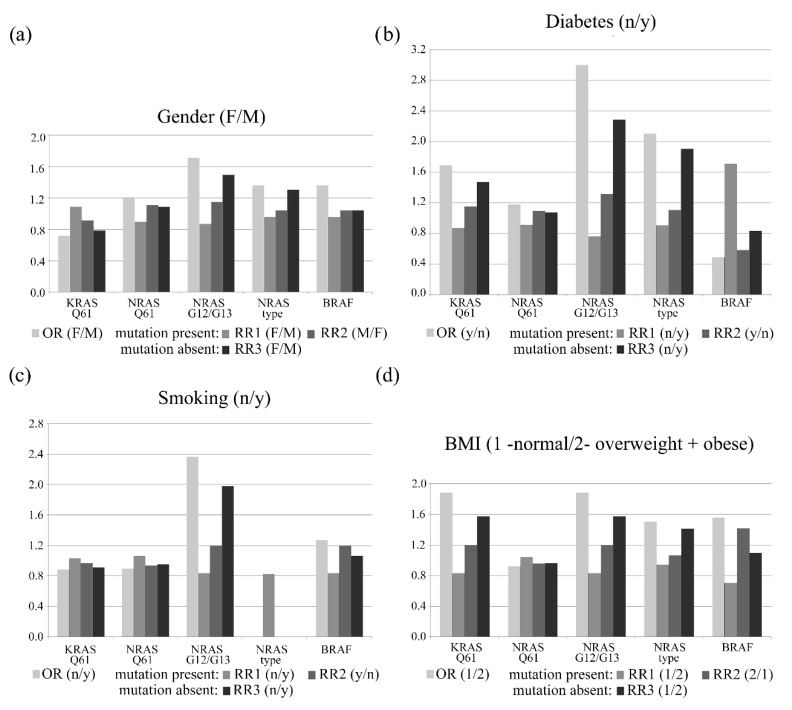
The association of the variables gender (**a**), diabetes (**b**), smoking (**c**), and BMI (**d**) with the presence of different mutation types illustrated by the values OR, RR1, RR2, and RR3 calculated for all possible combinations.

**Figure 3:**
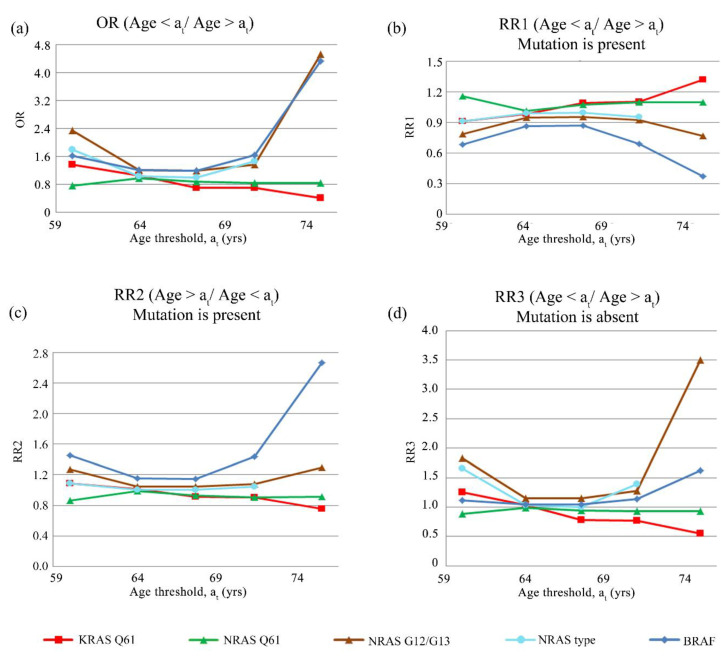
Association between age and mutational status. On the horizontal axis are presented the various threshold ages (a_t_) by which the cohort was divided into two groups: 1—patients younger than the threshold ages, and 2—patients older than the threshold ages. These values represent the percentiles 25%, 33%, 50% (or median), 67%, and 75% of the “age” variable. The association was illustrated by the calculated OR (**a**), RR1 (**b**), RR2 (**c**), and RR3 (**d**).

We calculated OR, RR1, RR2, and RR3 to associate different clinical data i.e., gender, diabetes, BMI groups (normal weight patients /overweight and obese patients), smoking, and age groups as risk factors for mutations (Figure 2 and Figure 3). We estimated these parameters in the following situations: Firstly, we considered the total absence of the mutation (*AF*‰ = 0) versus the cases corresponding to the mutations with 0.1 ≤ *AF*‰ < 1.0, and secondly, we considered the absence of the mutation (AF = 0) versus the cases corresponding to the mutations with *AF*‰ ≥ 1.0. In both situations, the risk estimates remained relatively constant. Thus, risk estimation was performed considering that *AF*‰ ≥ 0.1 corresponds to the presence of mutation.

The analysis of the association between gender and the mutational status revealed that the absence of NRAS G12/G13 mutations was associated with female gender (OR = 1.714, 95% CI = [0.528, 5.561] and RR3 = 1.495, 95% CI = [0.622, 3.590]) (Figure 2a). The absence of NRAS G12/G13 mutations (OR = 3.000, 95% CI = [0.879, 10.244] and RR3 = 2.286, 95% CI = [0.887, 5.887]), and of KRAS Q61 mutations (OR = 1.692, 95% CI = [0.534, 5.364] and RR3 = 1.469, 95% CI = [0.630, 3.429]) were associated with CRC tumors from non-diabetic patients. The presence of BRAF mutations (RR1 = 1.714, 95% CI = [0.657, 4.216]) was also associated with these CRC tumors. (Figure 2b).

The absence of NRAS G12/G13 mutations was found to be associated with tumors from non-smoking patients (OR = 2.364, 95% CI = 10.464, 12.048] and RR3 = 1.978, 95% CI = [0.506, 7.732]) (Figure 2c). CRC tumors from overweight and obese patients (BMI > 25.0) presented a relative risk for BRAF mutations (OR = 1.556, 95% CI = [0.369, 6.551] and RR2 = 1.416, 95% CI = [0.932, 1.900]). In normal weight patients, tumors exhibited associations with the absence of KRAS Q61 (OR = 1.879, 95% CI = [0.543, 6.499] and RR3 = 1.569, 95% CI = [0.662, 3.719]) and of the NRAS G12/G13 (OR = 1.879, 95% CI = [0.543, 6.499] and RR3 = 1.569, 95% CI = [0.662, 3.719]) mutations (Figure 2d). CRC in some age groups were significantly associated to BRAF and NRAS G12/G13 mutations (Figure 3a). Patients older than 75 years were more likely to have tumors that exhibit mutations in BRAF (OR = 4.333, 95% CI = [1.130, 16.612] and RR2 = 2.667, 95% CI = [2.457, 2.875]) (Figure 3b). Patients younger than 75 years old were more likely to have tumors that exhibit the absence of NRAS G12/G13 mutations (OR = 4.529, 95% CI = [0.533, 38.484] and RR3 = 3.500, 95% CI = [0.509, 24.056]) (Figure 3c).

### 3.3. Morphopathological Association with Mutations Presence

Systematic analysis in terms of the association of genetic mutations with histopathological phenotypes may be the premise of creating individualized treatments for CRC patients, more refined than current therapeutic solutions based on consensus on molecular subtypes.

#### 3.3.1. Tumor Localization

The presence of NRAS Q61 mutations was correlated to right colon tumor localization with OR = 4.160 (95% CI = [1.226, 14.1137]), and a RR2 = 2.755 (95% CI = [2.538, 2.970]), while the absence of NRAS Q61 mutations was associated with the left colon tumor localization by RR3 = 1.510 (95% CI = [1.042, 2.188]). Similar associations were found between the presence of NRAS G12/G13 mutations and right colon localization: OR = 3.482 (95% CI = 0.692, 17.515]) and RR2 = 2.618 (95% CI = [2.334, 2.900]), while the absence of this mutation was associated with the left colon localization RR3 = 1.331 (95% CI = [0.991, 1.788]). When considered together, the absence of NRAS type mutations was associated with left colon tumors RR2 = 1.515 (95% C1 = [1.242, 1.849]). Weaker associations were found between right colon tumors and other mutations, such as BRAF mutations OR = 1.939 (95% CI = [0.562, 6.698]) and RR2= 1.563 (95% CI = [1.209, 1.915]), and EGFR mutations, RR2 = 1.424 (95% CI = [1.314, 1.534]). For additional results, see Tables S3 and S4.

#### 3.3.2. Tumor Differentiation

The presence of KRAS Q61 mutations was strongly associated with the poorly differentiated tumors: OR = 1.842, (95% CI = [0.198, 17.179] and RR2 = 1.745, (95% CI = [1.245, 2.245]), while EGFR mutation presence was also associated with the same group RR2 = 1.727 (95% CI = [1.612, 1.842]). When NRAS Q61 or NRAS G12/G13 or BRAF mutations were missing, tumors were poorly differentiated RR1 = 4.355 (95% CI = [0.542, 35.002]) RR1 = 2.867 (95% CI = [0.647, 12.700]) and RR1 = 1.744 (95% CI = [0.221, 13.754]), respectively. All NRAS type mutations were associated to the group formed by the well and moderately differentiated tumors: RR2 = 1.504 (95% CI = [1.225, 1.781]). For additional results see Tables S3 and S4.

#### 3.3.3. HP Phenotypes

Extended necrosis was associated with NRAS Q61 mutations, OR = 2.670 (95% CI = [0.886, 8.046]) and RR2 = 1.866 (95% CI = [1.592, 2.138]); and EGFR mutations, RR2 = 1.582 (95% CI = [1.468, 1.696]). The mucoid phenotype was more strongly correlated with the presence of the EGFR mutations, RR2 = 4.739 (95% CI = [4.655, 4.823]) and with the absence of the NRAS Q61 mutations (RR1 = 2.903, 95% CI = [0.890, 9.472] or that of any NRAS-type mutations RR1 = 1.875 (95% CI = [0.655, 5.371]). The tumors in which the NRAS Q61 mutations were absent, were more likely to exhibit both glandular sub-types RR1 = 1.555 (95% CI = [0.915, 2.462]). We did not find any statistically significant association between the presence of the investigated mutations and the mixed tubular and cribriform glandular sub-phenotypes. For additional results see Tables S3 and S4.

#### 3.3.4. Limits of Invasion

Samples with the invasion limited by the submucosa were associated with the presence of NRAS Q61 mutations RR2 = 2.299 (95% CI = [1.905, 2.691]) and the presence of BRAF mutation RR2 = 5.747 (95% CI = [5.590, 5.509]). Samples from tumors with the invasion limit beyond the muscularis propria were associated with the presence of EGFR mutations RR2 = 1.424 (95% CI = [1.314, 1.534]). Samples with the invasion limit beyond the subserosa were related to the presence of KRAS Q61 mutations OR = 3.107 (95% CI = [0.923, 10.462]) and RR2 = 1.686 (95% CI = [1.401, 1.971]), and also to the EGFR mutations RR2 = 1.678 (95% CI = [1.563, 1.791]). In samples in which the tumor invaded beyond the *serosa*, the association with KRAS Q61 mutation decreased: OR = 1.630 (95% CI = [0.444, 5.984]) and RR2 = 1.395 (95% CI = [0.961, 1.827]), but the association with EGFR mutation presence increased RR2 = 3.003 (95% CI = [2.901, 3.105]). For additional results, see Tables S3 and S4.

#### 3.3.5. Desmoplastic Reaction

The KRAS Q61 mutations were associated with moderate and high desmoplastic reaction (OR = 4.222, 95% CI = [0.485, 36.767]; RR2 = 1.439, 95% CI = [1.077, 1.801]; RR3 = 2.933, 95% CI = [0.653, 13.183]), and these associations were even stronger in the case of high desmoplastic reaction (OR = 4.800, 95% CI = [0.459, 50.155]; RR2 = 2.725, 95% CI = [2.419, 3.031]; RR3 = 1.760, 95% CI = [0.934, 3.317]). Also, the presence of high-grade desmoplastic reaction was associated with the NRAS Q61 mutations (OR = 2.917, 95% CI = [0.594, 14.327]; RR3 = 1.719, 95% CI = [0.721, 4.098]; RR2 = 1.698, 95% CI = [1.381, 2.015]). For additional results see Tables S3 and S4.

#### 3.3.6. Lymphovascular and Perineural Invasion

Tumors with lymphovascular invasion were associated to KRAS Q61 OR = 2.177 (95% CI = [0.597, 7.933]), RR2 = 1.656 (95% CI = [1.295, 2.015], or to EGFR mutations RR2 = 2.591, (95% CI = [2.482, 2.698]). Perineural invasion was associated with the presence of NRAS Q61 mutations, OR = 1.705, (95% CI = [0.600, 4.849]), RR2 = 1.339, (95% CI = [1.012, 1.664], or of the EGFR mutations RR2 = 2.278, (95% CI = [2.165, 2.389]). Perineural invasion was also associated with the absence of BRAF mutations OR = 0.347, (95% CI = [0.095, 1.262]) and RR1 = 1.919, (95% CI = [0.789, 4.665]). For additional results see Tables S3 and S4.

#### 3.3.7. Other invasion Features

Budding of all grades was associated with NRAS-type mutations, OR = 3.542 (95% CI = [0. 752, 16.683]) and RR2 = 1.815 (95% CI = [1.484, 2.144]), and also to EGFR mutations, RR2 = 1.582 (95% CI = [1.468, 1. 696]). Adenomatous polyps were related to the following mutations: KRAS Q61, OR = 2.370 (95% CI = [0.580, 9.691), and RR2 = 1.859 (95% CI = [1.502, 2.214]); any of the NRAS type mutations OR = 1.545 (95% CI = [0.281, 8.493]) and RR2 = 1.361 (95% CI = [0.833, 1.887]); and EGFR, RR2 = 1.499 (95% CI = [1.387, 1.611]). The invasion in the close proximity of the tumor (liver, visceral, or parietal pleura, etc.) was associated with KRAS Q61, OR = 2.177 (95% CI = [0.557, 7.933]); RR2 = 1.656 (95% CI = [1.295, 2.015]); and EGFR mutation RR2 = 1.678 (95% CI = [1.563, 1.791]). This feature was also associated with the absence of any NRAS-type mutation RR1 = 1.736 (95% CI = [0.905, 3.331]) and that of BRAF mutations (RR1 = 1.657 (95% CI = [0.671, 4.092]).

#### 3.3.8. Inflammatory Infiltrate

The presence of the KRAS Q61 mutation was associated with the presence of mixed inflammatory infiltrate (OR = 4.889, 95% CI = [1.157, 20.665]; RR2 = 2.667, 95% CI = [2.424, 2.910]; RR3 = 1.833, 95 CI = [1.146, 2.934]). Regarding the quantitative differentiation of this infiltrate, the presence of the KRAS Q61 mutations (OR = 3.545, 95% CI = [0.683, 18.397]; RR2 = 2.597, 95% CI = [2.311, 2.883]) and the absence of NRAS mutations (RR1 = 1.792, 95% CI = [0.705, 4.556]) were associated with moderate or high inflammatory infiltrate. Along with the KRAS Q61 mutations (OR = 1.500, 95% CI = [0.271, 8.300], RR2= 1.401, 95% CI = [0.856, 1.946]; RR3 = 1.364, 95% CI = [0.978, 1.902]), the BRAF mutations were associated with a high-grade mixed inflammatory infiltrate (OR = 1.722, 95% CI = [0.358, 8.295]; RR2 = 1.541, 95% CI = [1.083, 1.999]), especially with lymphocyte-rich infiltrates (OR = 3.083, 95% CI = [0.179, 53.158]; RR2 = 2.924, 95% CI = [2.605, 3.243]). For additional results see Supplementary Materials Tables S3 and S4.

#### 3.3.9. ypTNM and AJCC Stagings

The group of ypT4-staged tumors and the group of ypT2-T3-staged tumors were associated with KRAS Q61 mutations (OR = 2.234, 95% CI = [0.434,11.504]; RR2 = 1.919, 95% CI = [1.528, 2.310]) and with the absence of the following mutations: BRAF (RR1 = 1.919, 95% CI = [0.479, 7.682]), any NRAS mutations (RR1 = 1.875, 95% CI = [0.655, 5.371]) i.e., NRAS Q61 (RR1 = 1.394, 95% CI = [0.517, 3.754]), and NRAS G12/G13 (RR1 = 1.792, 95% CI = [0.693, 4.634]).

The group of ypT2 tumors vs. the group of ypT3-T4 tumors was associated with the absence of KRAS Q61 (OR = 1.500, 95% CI = [0.246, 9.163] and RR3 = 1.433, 95% CI = [0.292, 7.045]), both NRAS-type mutations (OR = 3.833, 95% CI = [0.574, 25.595] and RR3 = 3.125, 95% CI = [0.680, 14.353]), i.e., NRAS Q61 (OR = 1. 852, 95% CI = [0.312, 11.008] and RR3 = 1.742, 95% CI = [0.346, 8.776]) and NRAS G12/G13 (OR = 3.333, 95% CI = [0.594, 18.717] and RR3 = 2.867, 95% CI = [0. 647, 12.700]). For additional results see Supplementary Materials Table S4.

The group of ypN2 vs. ypN0-N1 staged tumors was associated with the presence of KRAS Q61 mutation (OR = 2.270, 95% CI = [0.250, 20.582] and RR2 = 2.092, 95% CI = [1.677, 2.507]) and with the absence of NRAS-type mutations (RR1 = 2.500, 95% CI = [0.581, 10.766]), particularly with the absence of NRAS Q61 (RR1 = 2.177, 95% CI = [0.459, 10.326]). On the other hand, the group of ypN1-N2 vs. ypN0 was associated with the presence of the following mutations: KRAS Q61 (OR = 3.163, 95% CI = [0.869, 11.509] and RR2 = 2.004, 95% CI = [1.711, 2.297]) and EGFR (RR2 = 2.193, 95% CI = [2.080, 2.304]) and with the absence of BRAF mutation (RR1 = 1.535, 95% CI = [0.709, 3.325]) and of any NRAS-type mutations (RR1 = 1.420, 95% CI = [0.763, 2.644]).

Metastatic (M1) vs. non-metastatic (M0) status was associated with the presence of the KRAS Q61 mutations (OR = 9.154, 95% CI = [1.100, 76.175] and RR2 = 5.917, 95% CI = [5.772, 6.062]) and with the absence of any of the NRAS-type mutations (RR1= 1.786, 95% CI = [0.784, 4.068]), in particular the NRAS Q61 mutation (RR1 = 1.369, 95% CI = [0.618, 3.030]). For additional results see Supplementary Materials Table S4.

Likewise, the III-IV vs. I-II AJCC stages were related to the presence of the KRAS Q61 mutations (OR = 3.375, 95% CI = [0.978, 11.650] and RR2 = 1.883, 95% CI = [1.602, 2.164]) and to the absence of any of the NRAS-type mutations (RR1 = 1.442, 95% CI = [0.892, 2.332]). For additional results see Supplementary Materials Table S4.

Thus, histopathological analyses in conjecture with genetic ones can contribute to the revelation of disease current status and the identification of diagnostic and prognostic biomarkers that allow indications of personalized treatment.

## 4. Discussion

For the first time in a Romanian cohort, the CRC clinicopathological variables association with the mutational status of KRAS, NRAS, BRAF, and EGFR genes was explored. As these mutations are involved in the CRC development, and are currently considered as parameters to guide post-resection treatment decisions, we believe the findings we report here could contribute to refining post-resection therapy planning. We employed ddPCR, a very sensitive technique that allows absolute target quantification, and enabled us to assess the concomitant presence of low-frequency mutations.

**Figure 4:**
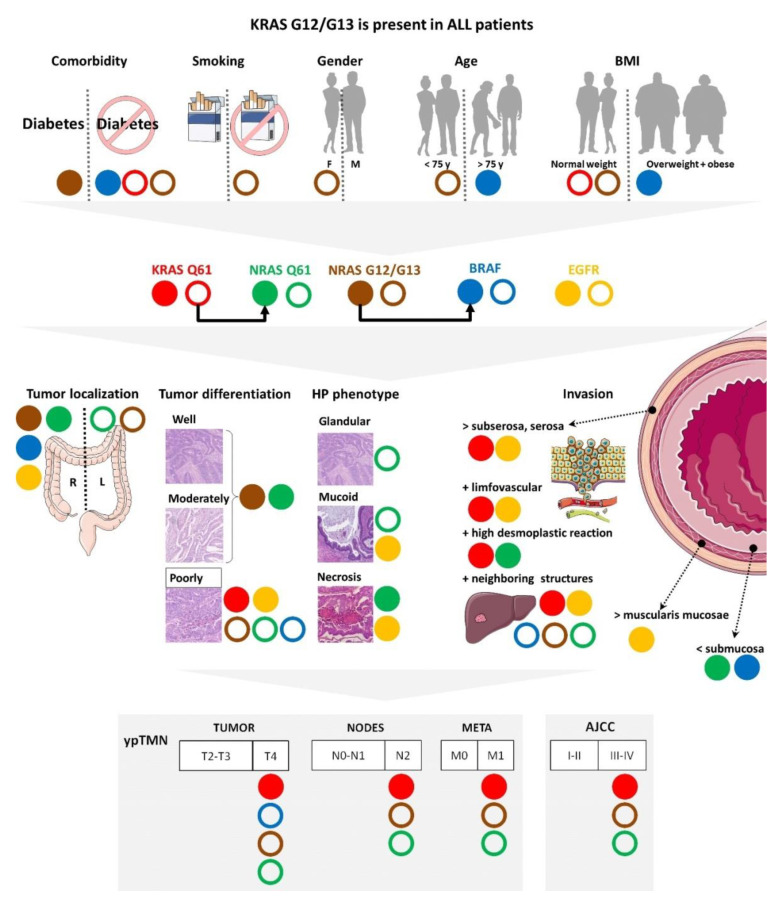
Integrative diagram of the most significant findings. These associations were evaluated considering the separate presence and absence of each of the studied mutations (labelled in different colors). The filled circles signify the association with the mutation’s presence (*AF*‰ ≥ 0.1), while the empty circles indicate the association with the mutation’s absence (*AF*‰ = 0). In the upper register, we considered that the mutation’s presence or absence is correlated with the absence or presence of a risk factor. The significant associations between the KRAS Q61 and NRAS Q61 mutations, on the one hand, and between the NRAS G12/G13 and BRAF mutations, on the other hand, are represented by the black arrows. Thus, the first black arrow shows that the absence of the KRAS Q61 mutation is significantly associated with the presence of the NRAS Q61 mutation. Also, the second black arrow shows that the coexistence of NRAS G12/G13 and BRAF mutations is statistically significant. In the lower register, we resumed the important associations between mutational status and the histopathological characteristics and CRC staging.

In our cohort, 96.7 % of CRC tumors harbored KRAS G12/G13 mutations and 21.1 % had KRAS Q61 mutations, both with *AF*‰ ≥ 1. All tumors that bore KRAS Q61 mutations had a KRAS G12/G13 mutations with an *AF*‰ ≥ 1. NRAS Q61 mutations were present in 15% of CRC tumors, while NRAS G12/G13 mutations were present in 20% with an *AF*‰ ≥ 1. Notably, only two tumors exhibited both of these mutations at the mentioned AFs. BRAF V600 mutations (*AF*‰ ≥ 1) were present in 11.7% of the tumors, and all these tumors exhibited KRAS G12/G13 mutations with an *AF*‰ averaging at 3.2. The percentage of BRAF V600 mutations in our cohort did not differ from that described in previous clinical studies, being associated with the right-side colon localization of the tumor [4,8,31,32]. Other authors reported incidences of KRAS G12/G13 mutations of approximately 30–50% [4,16,31], a lower value than reported by us. The high percentage of mutations in exon 2 of the KRAS gene we reported in our cohort could be explained by the quality of the isolated gDNA and the use of highly sensitive ddPCR. The KRAS G12/G13 kit we used allowed for the detection of all the frequent mutations encountered in exon 2 of the gene (G12A, G12C, G12D, G12R, G12S, G12V, and G13D), and thus any of these mutations in the same tumor sample, can contribute towards classification of the sample as being mutation positive. In addition, cellular and genetic tumor heterogeneity could be another relevant factor [3]. Our results demonstrate that BRAF and KRAS exon 2 mutations were not mutually exclusive and that their concomitant occurrence is not a rare event as claimed by other studies [4,17,33]. Additionally, several studies using paraffin-embedded tumor samples and detection technologies less sensitive than ddPCR reported the concomitant presence of KRAS and BRAF mutations, but in a lower percentage (below 4%) [34,35] than that found in our study. Moreover, in another study, BRAF mutational status was only screened in the KRAS wild-type tumor specimens, and for this reason, they found similar relationships with the clinicopathologic features for both mutations [17]. Our data reveals the co-occurrence of KRAS, NRAS, and BRAF mutations (Table 2 and Table 3) in CRC, which could explain why only a reduced number of patients (10–20%) respond to single-agent treatments based on anti-EGFR antibodies [36]. The risk of metastasis or progression in CRC requires a rigorous early assessment based on the correlated analyses of clinical factors (gender, BMI, age), genetic mutations, and histopathological characteristics. A recent study of KRAS mutations in exons 2, 3, and 4 in stage I–IV CRC patients concluded that exon 3 mutations predict the worst prognosis, and suggested that mutations of different KRAS exons should be analyzed separately [37]. Our results are in accordance with this study, as we highlight the strong association of the KRAS Q61 mutations with tumors, demonstrating histopathological features with an adverse impact on the disease prognosis (Figure 4). Thus, KRAS Q61 mutations were the only ones (amongst the mutations studied) associated with the ypT4 stage that were also associated with the absence of any NRAS and BRAF mutations (statistically significant). In addition, the association of KRAS Q61 mutation with poorly differentiated tumors strengthens this aspect (Figure 4, Table S4).

Along with the EGFR mutations, the KRAS Q61 mutations were also linked with invasiveness beyond the serosa, lymphovascular invasiveness, and with the invasion of neighboring structures. The invasion mechanism was strongly correlated to the desmoplastic reaction, which, in our samples, was strongly associated with KRAS Q61 mutations and also with the NRAS Q 61 mutations. Moreover, the KRAS Q61 mutations were associated with moderate or high inflammatory infiltrate in the absence of any of the NRAS mutations. Its absence and that of the NRAS type mutations were associated with ypT2-graded tumors. The presence of KRAS Q61 mutation and the absence of the NRAS type mutations were associated with the ypN2 and ypM1 stages. Thus, our results indicate that stage III–IV tumors, according to the AJCC classification, are strongly associated with the presence of KRAS Q61 mutations.

Our results also reveal unprecedented correlations between clinical or histological data on the one hand, and the NRAS mutations on the other hand. The NRAS Q61 mutations were associated with limited invasiveness up to the submucosal level. Its absence was associated with the mucoid and glandular phenotype (Figure 4, Table S4). In contrast, another study found an association between mucinous adenocarcinoma and KRAS mutations, but not with NRAS or BRAF mutations [16].

The NRAS type mutations seem to have an impact on tumor localization, with a higher risk towards the right colon. The absence of these mutations was, however, linked to a higher risk probability for left colon tumors. Both these associations are significant. A recent study showed that only tumor location in the right colon exhibited a significant correlation with KRAS and BRAF mutational status [8]. The KRAS G12/G13 mutation was ubiquitous and the NRAS G12/G13 mutation coexisted in the tumor samples we analyzed with the presence of BRAF mutation (*AF*‰ ≥ 1).

In addition, both mutations were associated with many clinical variables. The NRAS G12/G13 mutations were present in CRC tumors from diabetic patients and were absent in those of non-diabetics, normal-weight patients, and female patients, and also absent in CRC tumors resected from patients under 75 years of age (Figure 2 and Figure 3, Table S4).

As in the case of the NRAS Q61 mutation, the BRAF mutation correlates with tumor invasiveness limited to the submucosa and the right-side colon localization, the latter association being weaker than in the case of NRAS mutations (Figure 4). Our results show that the BRAF mutation is associated with lymphocytes-rich high-grade inflammatory infiltrates that correspond with CMS 1, characterized by immune infiltration and high BRAF mutation percentage [6]. The inflammatory microenvironment is an essential contributor in tumor progression [38]; thus, the association between the levels of specific biomolecules and AF of genetic mutations requires further investigation. In our group, the BRAF mutation was more likely to be present in patients younger than 75 years but older than 60 years and with a BMI > 25.0. In contrast with other studies [39,40], we did not find any association between the BRAF mutation and the presence of mucinous features and poor tumor differentiation, the latter being revealed to strongly associate with KRAS Q61 mutations (Figure 4). Such quantitative genetic analysis could identify a constellation of specific biomarkers allowing risk stratification of CRC patients, more precise diagnosis, and prognosis prediction through the correlation with relevant histopathological elements.

A recent study on a Moroccan colon cancer patients cohort showed that KRAS-mutated colon cancers were significantly associated with the female gender, vascular invasion, classical adenocarcinoma phenotype, moderately differentiated tumors, advanced TNM stage (III–IV), left colon tumor localization, and a higher incidence of distant metastases at the time of diagnostic [41]. This study agrees with our findings in the case of KRAS Q61 mutations and their association with advanced TNM stage (III–IV), with KRAS G12/G13 mutation being ubiquitous. In addition, the same study reported a connection between the NRAS-type mutations and less-extensive invasiveness, which is in agreement with our data (Figure 4). In our cohort, the presence of KRAS Q61 and the absence of BRAF- and NRAS-type mutation were associated with T4, albeit, other reports claim that concomitant KRAS- and BRAF-positive mutational status are more prevalent in T3 and T4 tumors [34]. By multivariate non-aprioristic approaches, Isnaldi et al. [4] identified two distinct clinical-mutational profiles. The first profile groups included older patients bearing a BRAF mutation with right-side tumors localization, agreeing with our results and previous works [8,39,42], and the second profile group consisted of younger female patients positive for KRAS and PIK3CA mutations. Our data do not support this latter profile since the only statistically significant association found in our cohort with the female gender was the absence of NRAS G12/13 mutations. In addition, since the KRAS G12/G13 mutations were ubiquitous in our samples, we cannot relate these mutations to any gender. Additionally, we did not find a significant correlation between this mutation and gender in the case of KRAS Q61 mutations either.

In a recent study on a Chinese CRC cohort, the authors concluded that an NRAS mutation is an independent prognostic marker for distant metastasis in stage I–III patients, with shorter metastasis-free intervals than *NRAS* wild-type patients [33]. In contrast, our data showed that stage III–IV tumors were correlated with the absence of NRAS-type mutation (Figure 4 and Table S4). Thus, given the contradictory studies, a range of validated biomarkers, particularly prognostic and predictive markers, are required for the advancement towards personalized cancer treatment [43].

## 5. Conclusions

We employed a pair-wise association approach to assess the correlation between several mutations (KRAS Q61, KRAS G12 / G13, NRAS Q61, NRAS G12 / G13, BRAF, and EGFR) and also the associations between the mutations and histopathology features (tumor staging, inflammation, differentiation, and invasiveness). The strongest associations we found and the mutational AF we reported may help to understand disease processes and may be considered as potential CCR biomarker candidates. In addition, we described representative mutation panels associated with specific clinical and histopathological features of CRC.

The KRAS Q61 mutations were associated with most of the invasive features of CRC described by histopathological variables (poor differentiation, microscopic and macroscopic invasiveness, and staging) with consequences on the disease prognosis (ypT4M1N2). The absence of NRAS types of mutation was associated with the same or with other histopathological features with different levels of impact on the aggressiveness of the disease. Our analysis revealed that KRAS Q61 and NRAS mutations have distinct clinical-pathological features, and KRAS G12/G13 mutation with different AFs was ubiquitous in this cohort, probably being essential for the CRC initiation and development.

Thus, our findings suggest refining the CRC consensus molecular subtypes classification by including other mutations such as KRAS Q61 and NRAS-type mutations and the AF levels of CRC-related mutations. Furthermore, based on the AF of the studied mutations, patient cohorts might be organized into different risk groups as per histopathological features. Such risk stratification opens up significant prospects for sensitive technologies such as ddPCR to be used in CCR screening and preventive, personalized treatment. As fresh tissue samples can be easily obtained by routine endoscopic investigations or during resection surgeries, a quantitative mutation analysis offers enormous potential to promote the future development of screening methods. This genetic analysis approach corroborated with histological observations could have a significant potential to indicate progression risk, thus guiding therapeutic indications for more effective treatments and increasing the cancer-free period and overall survival of CRC patients.

## Figures and Tables

**Table 1:** Descriptive statistics of mutation levels.

*AF*‰	0.0 ≤ *AF*‰ < 0.1		0.1 ≤ *AF*‰ < 1.0		*AF*‰ ≥ 1		*AF*‰ ≥ 0.1	
Mutation type	N	%	N	%	N	%	N	%
KRAS Q61	16	26.7	31	51.7	13	21.7	44	73.3
KRAS G12/G13	0	0.0	2	3.3	58	96.7	60	100.0
NRAS Q61	33	55.0	18	30.0	9	15.0	27	45.0
NRAS G12/G13	15	25.0	30	50.0	15	25.0	45	75.0
BRAF	45	75.0	8	13.3	7	11.7	15	25.0
EGFR	57	95.0	0	0.0	3	5.0	3	5.0

**Table 2:** The coexistence of KRAS G12/G13, KRAS Q61, NRAS G12/G13, NRAS Q61, BRAFV600, and EGFR exon 19 deletions mutations.

***AF*‰**	***AF*‰ ≥ 0.1**		***AF*‰ ≥ 1**	
Concomitant mutations	N	%	N	%
0	0	0.0	2	3.3
1	1	1.7	20	33.3
2	11	18.3	28	46.7
3	26	43.3	10	16.7
4	17	28.3		
5	5	8.3		
Total	60	100.0	60	100.0

**Table 3:** The contingency table presents the coexistence of genetic mutations: 0 represents the absence of mutation, and 1 the presence of the mutation (*AF*‰ ≥ 0.1).

		**KRAS G12/G13**		**NRAS Q61**		**NRAS G12/G13**		**BRAF**		**EGFR**	
0	1	0	1	0	1	0	1	0	1	0	1
**KRAS Q61**	0	0	16	7	9	4	12	12	4	14	2
		0%	26.7%	11.7%	15.0%	6.7%	20.0%	20.0%	6.7%	23.3%	3.3%
	1	0	44	26	18	11	33	33	11	43	1
		0%	73.3%	43.3%	30.0%	18.3%	55.0%	55.0%	18.3%	71.7%	1.7%
		**KRAS G12/G13**	0	0	0	0	0	0	0	0	0
				0%	0%	0%	0%	0%	0%	0%	0%
			1	33	27	15	45	45	15	57	3
				55.0%	45.0%	25.0%	75.0%	75.0%	25.0%	95.0%	5.0%
				**NRAS Q61**	0	8	25	25	8	30	3
						13.3%	41.7%	41.7%	13.3%	50.0%	5.0%
					1	7	20	20	7	27	0
						11.7%	33.3%	33.3%	11.7%	45.0%	0.0%
						**NRAS G12/G13**	0	14	1	15	0
								23.3%	1.7%	25.0%	0.0%
							1	31	14	42	3
								51.7%	23.3%	70.0%	5.0%
								**BRAF**	0	42	3
										70.0%	5.0%
									1	15	0
										25.0%	0.0%
										**EGFR**	0
										
											1
										

## Data Availability

The data supporting this study’s findings are available from the corresponding author, AIS, and the co-author and data curator LB upon reasonable request.

## Supplementary Materials

The following supporting information can be downloaded at: https://www.mdpi.com/article/10.3390/cancers14112792/s1, Figure S1: Risk analysis via OR, RR1, RR2, and RR3 of possible two-by-two associations between the studied mutations, as defined by Table S2; Table S1: Clinical and demographic data of the studied cohort; Table S2: Descriptive statistics of mutation levels (allelic frequencies) of all studied mutations; Table S3: Expressing associations between mutation levels via risk ratios RR1, RR2, and RR3; Table S4: Models used for OR, RR1, RR2, and RR3 calculation related to mutations status and tumor pathological features; Table S5: Risk estimation calculations using the models presented in Table S3.
