# Supplementary material for: Multivariate Risk Analysis of RAS, BRAF and EGFR Mutations Allelic Frequency and Coexistence as Colorectal Cancer Predictive Biomarkers
Source: Cancers (Basel). 2022 Jun 4;14(11):2792. doi: 10.3390/cancers14112792 (PMC9179415; doi:10.3390/cancers14112792)
Supplement: Supplementary file 1 [file cancers-14-02792-s001.zip › cancers-1732032-supplementary.pdf]

Article

Multivariate Risk Analysis of RAS, BRAF and EGFR Mutations Allelic Frequency and Coexistence as Colorectal Cancer Predictive Biomarkers

Adriana Ionescu, Liviu Bilteanu, Ovidiu Ionut Geicu, Florin Iordache, Loredana Stanca, Aurelia Magdalena Pisoschi, Adrian Miron, Andreea Iren Serban and Valentin Calu

Table S1. Clinical and demographic data of the studied cohort.

Variable	Mean	Median	Std. Deviation	Min.	Max.
**Age (yrs)**	**67.16**	**68.50**	**11.02**	**32**	**87**
Female	69.12	69.00	9.84	50	84
Male	65.54	66.00	11.44	22	87
**BMI (kg/m²)**	**26.74**	**26.52**	**3.97**	**18**	41
Female	27.18	26.30	4.55	19	41
Male	26.38	26.63	3.45	18	33
Variable	Category			Frequency	Percentage (%)
Sex	Female			26	43.3
	Male			34	56.7
BMI (kg/m²)	Underweight (<18.5)			1	1.7
	Normal weight (18.5 to 24.9)			16	26.7
	Overweight (≥ 25)			41	71.6
	Pre-obesity (25.0 to 29.9)			31	51.7
	Class 1 obesity (30.0 to 34.9)			9	15.0
	Class 2 obesity (30.0 to 34.9)			0	0
	Class 3 obesity (≥ 40)			1	1.7
	Unreported			2	NA

Diabetes	Diabetic patients	30	51.7
	Female	12	19.0
	Male	20	32.7
	Non-diabetics	28	48.3
	Female	14	24.1
	Male	14	24.1
Smoking	Smoking patients	12	20.7
	Female	2	4.5
	Male	11	18.2
	Non-smoking patients	46	79.3
	Female	24	41.4
	Male	22	37.9
Tumor localization	Left (L)	41	70.7
	Right (R)	17	29.3
ypT stage	T2	6	10.3
	T3	39	67.2
	T4	13	22.4
ypN stage	N0	31	53.4
	N1	20	34.5
	N2	7	12.1
ypM stage	M0	40	69.0
	M1	18	31.0
AJCC Staging	I	5	8.6
	IIA	18	31.0
	IIB	2	3.4
	IIIA	2	3.4
	IIIB	1	1.7
	IIIC	8	13.8

IV	20	34.5
Unreported	2	NA

Unreported cases are not included in the percentage calculations.

Table S2. Descriptive statistics of mutation levels (allelic frequencies) of all studied mutations

ALL cases included							
		**KRAS Q61**	**KRAS G12/G13**	**NRAS Q61**	**NRAS G12/G13**	**BRAF**	**EGFR**
LINEAR SCALE							
Mean		0.72	75.76	2.81	14.84	21.58	0.23
95% Confidence Interval for Mean	Lower Bound	0.57	40.74	-0.56	-0.72	4.52	-0.15
	Upper Bound	0.87	110.77	6.18	30.39	38.65	0.60
5% Trimmed Mean		0.69	58.35	0.45	2.59	9.84	0.00
Median		0.82	4.95	0.00	0.76	0.00	0.00
Variance		0.34	18377.06	170.16	3624.54	4362.42	2.09
Std. Deviation		0.58	135.56	13.04	60.20	66.05	1.44
Minimum		0.00	0.25	0.00	0.00	0.00	0.00
Maximum		3.48	599.63	85.48	342.30	266.46	11.07
Range		3.48	599.38	85.48	342.30	266.46	11.07
Interquartile Range		0.96	86.94	0.86	0.88	0.04	0.00
Skewness		1.51	1.98	5.67	4.70	3.05	7.44
Kurtosis		7.30	3.44	32.42	22.25	7.97	56.54
Percentiles							
25		0.65	2.78	0.00	0.14	0.00	0.00
33		0.82	3.17	0.00	0.65	0.00	0.00
50		0.90	4.95	0.00	0.76	0.00	0.00
67		0.96	10.67	0.76	0.96	0.00	0.00

		KRAS Q61	KRAS G12/G13	NRAS Q61	NRAS G12/G13	BRAF	EGFR
75		0.00	89.72	0.86	1.02	0.12	0.00
**Mutation-free cases excluded**							
		**KRAS Q61**	**KRAS G12/G13**	**NRAS Q61**	**NRAS G12/G13**	**BRAF**	**EGFR**
LINEAR SCALE							
Mean		0.99	75.76	6.25	19.78	92.50	4.51
95% Confidence Interval for Mean	Lower Bound	0.85	40.74	-1.30	27.3401	24.68	-9.66
	Upper Bound	1.12	110.77	13.79	157.6542	147.99	18.68
5% Trimmed Mean		0.93	58.35	2.58	5.52	87.96	0.00
Median		0.88	4.95	0.87	0.87	22.53	1.74
Variance		0.20	18377.06	363.86	4760.14	12734.88	32.54
Std. Deviation		0.44	135.56	19.08	68.99	112.885	5.70
Minimum		0.56	0.25	0.53	0.56	0.12	0.72
Maximum		3.48	599.63	85.48	342.30	266.46	11.07
Range		2.92	599.38	84.95	341.74	266.34	10.35
Interquartile Range		0.34	86.94	0.93	0.46	233.56	.
Skewness		4.33	1.98	3.71	4.01	.643	1.67
Kurtosis		23.73	3.44	13.35	15.94	-1.577	.
Percentiles							
25		0.80	2.78	0.73	0.71	0.26	0.72
33		0.82	3.17	0.77	0.75	0.32	1.05
50		0.88	4.95	0.87	0.87	22.53	1.74
67		0.96	10.67	1.02	1.02	172.98	8.08
75		1.15	89.72	1.66	1.17	233.82	
=		**KRAS Q61**	**KRAS G12/G13**	**NRAS Q61**	**NRAS G12/G13**	**BRAF**	**EGFR**
LOG SCALE							

Mean		-0.03	1.04	0.12	0.16	0.81	0.38
95% Confidence Interval for Mean	Lower Bound	-0.07	0.81	-0.09	-.0438	-0.18	-1.12
	Upper Bound	0.01	1.27	0.33	1.6555	1.51	1.89
5% Trimmed Mean		-0.04	1.03	0.05	0.05	0.81	.
Median		-0.06	0.69	-0.06	-0.06	0.75	0.24
Variance		0.02	0.80	0.28	0.47	2.16	0.37
Std. Deviation		0.13	0.89	0.53	0.68	1.47	0.61
Minimum		-0.25	-0.60	-0.28	-0.25	-0.92	-0.14
Maximum		0.54	2.78	1.93	2.53	2.43	1.04
Range		0.79	3.38	2.21	2.79	3.35	1.19
Interquartile Range		0.15	1.50	0.36	0.22	2.97	.
Skewness		1.85	0.64	2.80	2.83	0.00	0.99
Kurtosis		6.79	-0.84	7.65	6.91	-2.18	.
Percentiles							
25		-0.10	0.44	-0.14	-0.15	-0.60	-0.14
33		-0.09	0.50	-0.11	-0.12	-0.50	-0.02
50		-0.06	0.69	-0.06	-0.06	0.75	0.24
67		-0.02	1.03	0.01	0.01	2.24	0.79
75		0.06	1.94	0.22	0.07	2.37	

Coexistence of mutations and their associations with clinical and histopathological data

How mutations are mutually influencing each other, the so-called coexistence of mutations was determined by calculating the OR- and RR-type parameters in all possible situations where one of the mutations is considered a determining factor (determinant variable) for the other mutations that were considered dependent on this mutation (outcome variables). We considered the following situations:

1. Coexistence of mutations, regardless of AF‰. We thus distinguish for each mutation two cases: absence (encoded by the symbol 0) and presence (encoded by the symbol 1). In short, we have the following situations for each mutation X,

$$X = \begin{cases} 0, \text{if } AF(X) = 0 \\ 1, \text{if } AF(X) \geq 0.1 \end{cases} \tag{S1}$$

So, if X is the determinant mutation and Y is the outcome variable, we denote by X = 0 if the mutation X is absent and by X = 1 if the mutation X is present. By X or Y, we mean one of the studied mutations, KRAS Q61, KRAS G12 / G13, NRAS Q61, NRAS G12 / G13 or BRAF.

2. Coexistence of mutations taking into account AF‰ values. In this case, we have the following notations:

$$X = \begin{cases} 0, \text{if } AF(X) = 0 \\ 1, \text{if } 0.1 \leq AF(X) < 1 \\ 2, \text{if } AF(X) \geq 1 \end{cases} \tag{S2}$$

in the first case (in which we first considered that a mutation is present if $AF \geq 0.1$), we calculated the following coefficients:

Odds-ratio (OR) is invariable at the change X → Y and is calculated as the ratio of the following probabilities

$$OR = \frac{\frac{P(|Y=0|X=0)}{P(|Y=1|X=0)}}{\frac{P(|Y=0|X=1)}{P(|Y=1|X=1)}} = \frac{\frac{P(|Y=1|X=1)}{P(|Y=0|X=1)}}{\frac{P(|Y=1|X=0)}{p(|Y=0|X=0)}} \tag{S3}$$

where $P(|Y=0|X=0)$ is the relative probability or frequency of cases for which Y = 0 (Y mutation is absent), knowing that X = 0 (X mutation is present).

Relative risk (RR_1) is calculated as:

$$RR_1 = \frac{P(Y=1|X=0)}{P(Y=1|X=1)} \quad \text{(S4)}$$

The risk of Y mutation in cases X = 0 compared to cases X = 1. If $RR_1 > 1$ or more precisely $RR_1 \geq 1.3$, then the cases X = 0 (i.e. the cases in which the determinant mutation X is absent) have RR1 times higher relative risk than the cases X = 1 (when the mutation X is present) to be associated with the presence of the Y mutation (Y = 1). In short, the absence of the X mutation increases by RR1 folds the risk of the Y mutation occurrence compared to the absence of X mutation.

Relative risk (RR_2) calculated as:

$$RR_2 = \frac{P(Y=1|X=1)}{P(Y=1|X=0)} \quad \text{(S5)}$$

that is, the risk of Y mutation in cases X = 1 compared to cases X = 0. If $RR_2 > 1$ (or more precisely $RR_2 > 1.3$), then the cases X = 1 (in which the determinant mutation X is present) have RR_2 folds higher relative risk than the cases X = 0 (when the mutation X is absent) to be associated with the presence of the Y mutation (Y = 1). In other words, the presence of the X mutation increases by RR_2 folds the risk of the Y mutation occurring if X is absent.

Relative risk (RR_3) calculated as

$$RR_3 = \frac{P(Y=0|X=0)}{P(Y=0|X=1)} \quad \text{(S6)}$$

that is, the risk of Y mutation absence in cases X = 0 compared to cases X = 1. If $RR_3 > 1$ (or more precisely$RR_3 > 1.3$), then the cases X = 0 (in which the determinant mutation X is absent) have RR_3 folds higher relative risk than the cases when the mutation X is present (X = 1) to be associated with the absence of the Y mutation (Y = 0). In other words, the absence of the X mutation increases by RR_3 folds the risk of the Y mutation absence compared to X mutation presence.

We have the following equations between the four parameters

$$RR_1 \times RR_2 = 1$$
$$RR_1 \times RR_3 = OR \quad \text{(S7)}$$

The same coefficients OR, RR1 and RR3 were calculated for all possible combinations (X = {0, 1, 2} | Y = {0, 1, 2}) were also calculated in the second case.

Risk factor - mutation association is expressed by the same type of coefficients. The variable Y is associated with the mutation's absence (Y = 0) or presence (Y = 1) in the above formulas. The variable X will be one of the clinical variables: sex, X → {F, M}; diabetes, X → {no, yes} in which *no*- non diabetic patients, *yes* - diabetic patients; smoking: X → {no, yes}, BMI: X - {1, 2} where *1*- underweight + normal weight, *2*- overweight and obese patients. The meaning of the parameters OR, RR1, RR 2 and RR3 is the same as above. We used the OR and RR parameters defined above to study the association of a mutation as a risk factor for the values of tumour-related variables (laterality, histopathological features, tumour staging) listed in Table S3. In this case, the variable X in the above equations represents the presence or absence of one of the mutations, and Y represents one of the variables listed with the possible values, as shown in Table S3. A significant association between a clinical or a histopathological variable and a mutation will be considered when RR> 1.3.

Table S3. Expressing associations between mutation levels via risk ratios RR1, RR2 and RR3

Independent mutations values	Outcome mutation values	RR1 > 1.3	RR2 > 1.3	RR3 > 1.3

X→{0,1}<br>The cases with AF = 0 or 0.1 ≤ AF <1 for both X and Y mutations	Y→{0,1}	The absence of the X mutation determines the presence of 0.1 ≤ AF <1 levels of the Y mutation preferentially.	The presence of 0.1 ≤ AF <1 levels of the X mutation determines the presence of AF < 1 levels of the Y mutation preferentially.	The X mutation's absence determines the Y mutation's absence rather than the presence of 0.1 ≤ AF <1 levels of this mutation.
X→{0,1} i.e.<br>The cases with AF = 0 or 0.1 ≤ AF <1	Y→{0,2} i.e.<br>The cases with AF = 0 or AF ≥ 1	The absence of the X mutation determines the presence of AF ≥ 1 levels of Y mutation preferentially.	The presence of 0.1 ≤ AF <1 levels of the X mutation determines the presence of AF ≥ 1 levels of Y mutation preferentially.	The X mutation's absence determines the Y mutation's absence.
X→{0,1} i.e.<br>The cases with AF = 0 or 0.1 ≤ AF <1	Y→{1,2} i.e.<br>The cases with 0.1 ≤ AF <1 or AF ≥ 1	The absence of the X mutation determines the presence of AF ≥ 1 levels of Y mutation preferentially.	The presence of 0.1 ≤ AF <1 levels of the X mutation determines the presence of AF ≥ 1 levels of Y mutation preferentially.	The X mutation's absence determines the Y mutation's presence of 0.1 ≤ AF <1 levels preferentially.
X→{0,2} i.e.<br>The cases with AF = 0 or AF ≥ 1	Y→{0,1} i.e.<br>The cases with AF = 0 or 0.1 ≤ AF <1	The absence of the X mutation determines the presence of 0.1 ≤ AF <1	The presence of AF ≥ 1 levels of the X mutation determines the presence	The X mutation's absence determines the absence of the Y mutation preferentially.

		levels of the Y mutation preferentially.	of 0.1 ≤ AF <1 levels preferentially.	
X→{0,2} i.e<br>The cases with AF = 0 or AF ≥ 1	Y→{0,2} i.e.	The absence of the X mutation determines the presence of AF ≥ 1 levels of the Y mutation preferentially.	The presence of AF ≥ 1 levels of the X mutation determines the presence of AF ≥ 1 levels of the Y mutation preferentially.	The X mutation's absence determines the absence of the Y mutation preferentially.
X→{0,2} i.e.<br>The cases with AF = 0 or AF ≥ 1	Y→{1,2} i.e.<br>The cases with 0 < AF < 1 or AF ≥ 1	The absence of the X mutation determines the presence of AF ≥ 1 levels of Y mutation preferentially.	The presence of 0.1 ≤ AF <1 levels of the X mutation determines the presence of AF ≥ 1 levels of Y mutation preferentially.	The absence of the X mutation determines the presence of 0.1 ≤ AF <1 levels of Y mutation preferentially.
X→{1,2} i.e.<br>The cases with 0.1 ≤ AF <1 or AF ≥ 1	Y→{0,1} i.e.<br>The cases with AF = 0 or 0.1 ≤ AF <1	The presence of AF < 1 levels of the X mutation determines the presence of 0.1 ≤ AF <1 levels of the Y-mutation preferentially.	The presence of AF ≥ 1 levels of the X mutation determines the presence of 0.1 ≤ AF <1 levels of Y mutation preferentially.	The presence of 0.1 ≤ AF <1 levels of the X mutation determines the absence of the Y-mutation preferentially.
X→{1,2} i.e.<br>The cases with 0.1 ≤ AF <1 or AF ≥ 1	Y→{0,2} i.e.<br>The cases with AF = 0 or AF ≥ 1	The presence of 0.1 ≤ AF <1 levels of the X	The presence of AF ≥ 1 levels of the X mutation	The presence of 0.1 ≤ AF <1 levels of the X

		mutation determines the presence of AF ≥ 1 levels of Y mutation preferentially.	determines the presence of AF ≥ 1 levels of Y mutation than the absence of the Y-mutation preferentially.	mutation determines the absence of the Y-mutation preferentially.
X→{1,2}	Y→{1,2}	The presence of 0.1 ≤ AF <1 levels of the X mutation determines the presence of AF ≥ 1 levels of Y-mutation preferentially.	The presence of AF ≥ 1 levels of the X mutation determines the presence of AF ≥ 1 levels of Y-mutation preferentially.	The presence of 0.1 ≤ AF <1 levels of the X mutation determines the presence of 0.1 ≤ AF <1 levels of the Y-mutation preferentially.
The cases with 0.1 ≤ AF <1 or AF ≥ 1				

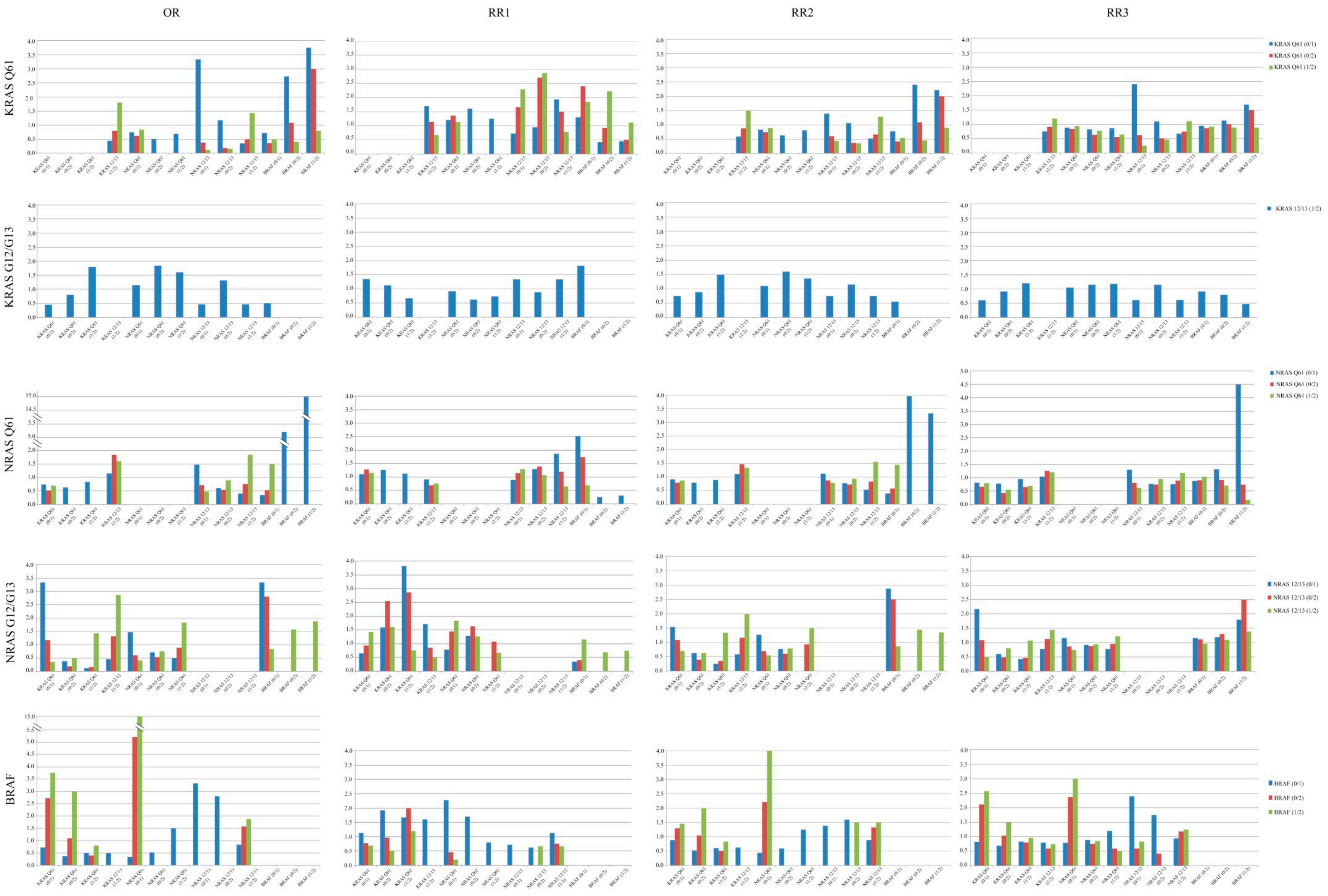
OR
RR1
RR2
RR3
KRAS Q61
KRAS G12/G13
NRAS Q61
NRAS G12/G13
BRAF
KRAS Q61 (0/1)
KRAS Q61 (0/2)
KRAS Q61 (1/2)
KRAS 12/13 (1/2)
NRAS Q61 (0/1)
NRAS Q61 (0/2)
NRAS Q61 (1/2)
NRAS 12/13 (0/1)
NRAS 12/13 (0/2)
NRAS 12/13 (1/2)
BRAF (0/1)
BRAF (0/2)
BRAF (1/2)

Figure S1. Risk analysis via OR, RR1, RR2 and RR3 of possible two-by-two associations between the studied mutations, defined according to Table S3

Table S4. Models used for OR, RR1, RR2 and RR3 calculation related to mutations status and tumour pathological features

1 – tumour invasion limited by the submucosa,
2 – tumour invasion beyond the submucosa;

Limits of invasion model 2

1 – tumour invasion limited by the muscularis mucosa,
2 – tumour invasion beyond the muscularis mucosa;

Limits of invasion model 3

1 – tumour invasion limited by the subserosa,
2 – tumour invasion beyond the muscularis subserosa;

Limits of invasion model 4

1 – tumour invasion limited by the serosa,
2 – tumour invasion beyond the muscularis serosa;

Desmoplastic reaction

Desmoplastic reaction Model1

1 – "low"; 2 – "moderate and high"

Desmoplastic reaction Model2

1 – "low and moderate"; 2 – "high"

Lymphovascular invasion, perineural invasion, adenomatous polyps or budding

0 – absent; 1 – present;

Inflammatory infiltrates

Tumour localization / laterality

L – left colon; R – right colon;

ALL mutations

0 – the mutation is absent, 1 – the mutation is present

Tumour differentiation grade

Differentiation grade grouping model 1

1 – well-differentiated / 2 - moderately and poorly differentiated tumours

Differentiation grade grouping model 2

1 – well and moderately / 2 - poorly differentiated tumours

Histopathological pattern

Tubular pattern

0 – absent, 1 – present

Cribriform pattern

0 – absent, 1 – present

Necrosis

0 – absent, 1 – present

Mucoid phenotype

0 – absent, 1 – present

Invasion limits

Limits of invasion model 1

ypT Model 1

1 – ypT2 or ypT3; 2 – ypT4

ypT Model 2

1 – ypT2; 2 – ypT3 or ypT4

ypN Staging

ypN Model 1

1 – ypN0 or ypN1; 2 – ypN2

ypN Model 2

1 – ypN0; 2 – ypN1 or ypN2

ypM Staging

M0 – no metastasis; M1 – metastasis in any organ;

AJCC Staging

1 –stage II CRC; 2 – stages III and IV CRC;

Mixed peri- and/or intratumoral infiltrate Model 1

1 – "absent"; 2 – "present (grade-independent) "

Mixed peri- and/or intratumoral infiltrate Model 2

1 – "absent or low"; 2 – "moderate or high"

Mixed peri- and/or intratumoral infiltrate Model 3

1 – "absent or low or moderate"; 2 – "high"

Lymphocyte peri- and/or intratumoral Model 1

1 – "absent"; 2 – "present (grade-independent)"

Lymphocyte peri- and/or intratumoral Model 2

1 – "absent or low"; 2 – "moderate or high"

Lymphocyte peri- and/or intratumoral Model 3

1 – "absent or low or moderate"; 2 – "high"

ypT Staging

Table S5. Risk estimation calculations using the models presented in Table S3. Parameters OR, RR1, RR2 and RR3 have been defined above.

Independent variable (Mutation)	Outcome Variable	Symbol	Value	95% IC LB	95% IC UB
KRAS Q61 (0/1)	Tumor Localization (left/right)	OR	1.192	0.319	4.445
KRAS Q61 (0/1)	Tumor Localization (left/right)	RR1	0.882	0.340	2.290
KRAS Q61 (0/1)	Tumor Localization (left/right)	RR2	1.134	0.592	1.676
KRAS Q61 (0/1)	Tumor Localization (left/right)	RR3	1.051	0.731	1.511
KRAS Q61 (0/1)	HP differentiation model 1 (1/2)	OR	0.632	0.184	2.162
KRAS Q61 (0/1)	HP differentiation model 1 (1/2)	RR1	1.194	0.765	1.865
KRAS Q61 (0/1)	HP differentiation model 1 (1/2)	RR2	0.838	0.409	1.267
KRAS Q61 (0/1)	HP differentiation model 1 (1/2)	RR3	0.754	0.342	1.663
KRAS Q61 (0/1)	HP differentiation model 2 (1/2)	OR	1.842	0.198	17.179
KRAS Q61 (0/1)	HP differentiation model 2 (1/2)	RR1	0.573	0.073	4.521
KRAS Q61 (0/1)	HP differentiation model 2 (1/2)	RR2	1.745	1.245	2.245

KRAS Q61 (0/1)	HP differentiation model 2 (1/2)	RR3	1.056	0.888	1.256
KRAS Q61 (0/1)	For HP phenotype with tubular pattern (0/1)	OR	0.696	0.072	6.773
KRAS Q61 (0/1)	For HP phenotype with tubular pattern (0/1)	RR1	1.029	0.872	1.215
KRAS Q61 (0/1)	For HP phenotype with tubular pattern (0/1)	RR2	0.972	0.815	1.129
KRAS Q61 (0/1)	For HP phenotype with tubular pattern (0/1)	RR3	0.717	0.087	5.917
KRAS Q61 (0/1)	HP phenotype with cribriform pattern (0/1)	OR	0.632	0.184	2.162
KRAS Q61 (0/1)	HP phenotype with cribriform pattern (0/1)	RR1	1.194	0.765	1.865
KRAS Q61 (0/1)	HP phenotype with cribriform pattern (0/1)	RR2	0.838	0.409	1.267
KRAS Q61 (0/1)	HP phenotype with cribriform pattern (0/1)	RR3	0.754	0.342	1.663
KRAS Q61 (0/1)	Necrosis (0/1)	OR	0.552	0.166	1.829
KRAS Q61 (0/1)	Necrosis (0/1)	RR1	1.433	0.718	2.861
KRAS Q61 (0/1)	Necrosis (0/1)	RR2	0.698	-0.017	1.413
KRAS Q61 (0/1)	Necrosis (0/1)	RR3	0.791	0.472	1.326
KRAS Q61 (0/1)	Limits of invasion model 1 (1/2)	OR	1.464	0.123	17.415
KRAS Q61 (0/1)	Limits of invasion model 1 (1/2)	RR1	0.979	0.842	1.138
KRAS Q61 (0/1)	Limits of invasion model 1 (1/2)	RR2	1.021	0.884	1.158
KRAS Q61 (0/1)	Limits of invasion model 1 (1/2)	RR3	1.433	0.140	14.693
KRAS Q61 (0/1)	Limits of invasion model 2 (1/2)	OR	1.292	0.365	4.570
KRAS Q61 (0/1)	Limits of invasion model 2 (1/2)	RR1	0.925	0.618	1.384
KRAS Q61 (0/1)	Limits of invasion model 2 (1/2)	RR2	1.081	0.774	1.388
KRAS Q61 (0/1)	Limits of invasion model 2 (1/2)	RR3	1.194	0.504	2.828
KRAS Q61 (0/1)	Limits of invasion model 3 (1/2)	OR	3.107	0.923	10.462
KRAS Q61 (0/1)	Limits of invasion model 3 (1/2)	RR1	0.593	0.308	1.140
KRAS Q61 (0/1)	Limits of invasion model 3 (1/2)	RR2	1.686	1.401	1.971
KRAS Q61 (0/1)	Limits of invasion model 3 (1/2)	RR3	1.843	1.015	3.346
KRAS Q61 (0/1)	Limits of invasion model 4 (1/2)	OR	1.630	0.444	5.984
KRAS Q61 (0/1)	Limits of invasion model 4 (1/2)	RR1	0.717	0.284	1.807
KRAS Q61 (0/1)	Limits of invasion model 4 (1/2)	RR2	1.395	0.962	1.828

KRAS Q61 (0/1)	Limits of invasion model 4 (1/2)	RR3	1.168	0.797	1.712
KRAS Q61 (0/1)	Desmoplasic Reaction Model 1	OR	4.222	0.485	36.767
KRAS Q61 (0/1)	Desmoplasic Reaction Model 1	RR1	0.695	0.333	1.448
KRAS Q61 (0/1)	Desmoplasic Reaction Model 1	RR2	1.439	1.077	1.801
KRAS Q61 (0/1)	Desmoplasic Reaction Model 1	RR3	2.933	0.653	13.183
KRAS Q61 (0/1)	Desmoplasic Reaction Model 2	RR1	0.367	0.061	2.205
KRAS Q61 (0/1)	Desmoplasic Reaction Model 2	RR2	2.725	2.419	3.031
KRAS Q61 (0/1)	Mixed peri- and/or intratumoral infiltrate Model 1	OR	4.889	1.157	20.665
KRAS Q61 (0/1)	Mixed peri- and/or intratumoral infiltrate Model 1	RR1	0.375	0.132	1.064
KRAS Q61 (0/1)	Mixed peri- and/or intratumoral infiltrate Model 1	RR2	2.667	2.424	2.910
KRAS Q61 (0/1)	Mixed peri- and/or intratumoral infiltrate Model 1	RR3	1.833	1.146	2.934
KRAS Q61 (0/1)	Mixed peri- and/or intratumoral infiltrate Model 2	OR	3.545	0.683	18.397
KRAS Q61 (0/1)	Mixed peri- and/or intratumoral infiltrate Model 2	RR1	0.385	0.099	1.489
KRAS Q61 (0/1)	Mixed peri- and/or intratumoral infiltrate Model 2	RR2	2.597	2.311	2.883
KRAS Q61 (0/1)	Mixed peri- and/or intratumoral infiltrate Model 3	OR	1.500	0.271	8.300
KRAS Q61 (0/1)	Mixed peri- and/or intratumoral infiltrate Model 3	RR1	0.714	0.169	3.027
KRAS Q61 (0/1)	Mixed peri- and/or intratumoral infiltrate Model 3	RR2	1.401	0.856	1.946
KRAS Q61 (0/1)	Mixed peri- and/or intratumoral infiltrate Model 3	RR3	1.071	0.817	1.404
KRAS Q61 (0/1)	Mixed peri- and/or intratumoral infiltrate Model 2	RR3	1.364	0.978	1.902
KRAS Q61 (0/1)	Lymphocyte peri- and/or intratumoral Model 1	OR	0.745	0.219	2.531
KRAS Q61 (0/1)	Lymphocyte peri- and/or intratumoral Model 1	RR1	1.137	0.679	1.904
KRAS Q61 (0/1)	Lymphocyte peri- and/or intratumoral Model 1	RR2	0.880	0.422	1.338
KRAS Q61 (0/1)	Lymphocyte peri- and/or intratumoral Model 1	RR3	0.847	0.417	1.722
KRAS Q61 (0/1)	Lymphocyte peri- and/or intratumoral Model 2	OR	0.400	0.100	1.599
KRAS Q61 (0/1)	Lymphocyte peri- and/or intratumoral Model 2	RR1	2.000	0.719	5.561
KRAS Q61 (0/1)	Lymphocyte peri- and/or intratumoral Model 2	RR2	0.500	-0.781	1.781
KRAS Q61 (0/1)	Lymphocyte peri- and/or intratumoral Model 2	RR3	0.800	0.544	1.177
KRAS Q61 (0/1)	Lymphocyte peri- and/or intratumoral Model 3	OR	0.400	0.023	6.848

KRAS Q61 (0/1)	Lymphocyte peri- and/or intratumoral Model 3	RR1	2.400	0.160	35.911
KRAS Q61 (0/1)	Lymphocyte peri- and/or intratumoral Model 3	RR2	0.417	-1.823	2.657
KRAS Q61 (0/1)	Lymphocyte peri- and/or intratumoral Model 3	RR3	0.960	0.830	1.111
KRAS Q61 (0/1)	PMN peri- and/or intratumoral infiltrate Model 1	OR	0.783	0.225	2.723
KRAS Q61 (0/1)	PMN peri- and/or intratumoral infiltrate Model 1	RR1	1.167	0.540	2.522
KRAS Q61 (0/1)	PMN peri- and/or intratumoral infiltrate Model 1	RR2	0.857	0.230	1.484
KRAS Q61 (0/1)	PMN peri- and/or intratumoral infiltrate Model 1	RR3	0.913	0.566	1.472
KRAS Q61 (0/1)	PMN peri- and/or intratumoral infiltrate Model 2	OR	0.500	0.129	1.940
KRAS Q61 (0/1)	PMN peri- and/or intratumoral infiltrate Model 2	RR1	1.667	0.628	4.420
KRAS Q61 (0/1)	PMN peri- and/or intratumoral infiltrate Model 2	RR2	0.600	-0.439	1.639
KRAS Q61 (0/1)	PMN peri- and/or intratumoral infiltrate Model 2	RR3	0.833	0.562	1.236
KRAS Q61 (0/1)	PMN peri- and/or intratumoral infiltrate Model 3	OR	0.242	0.036	1.633
KRAS Q61 (0/1)	PMN peri- and/or intratumoral infiltrate Model 3	RR1	3.500	0.650	18.852
KRAS Q61 (0/1)	PMN peri- and/or intratumoral infiltrate Model 3	RR2	0.286	-2.564	3.136
KRAS Q61 (0/1)	PMN peri- and/or intratumoral infiltrate Model 3	RR3	0.848	0.650	1.107
NRAS Q61 (0/1)	Tumor Localization (left/right)	OR	4.160	1.226	14.113
NRAS Q61 (0/1)	Tumor Localization (left/right)	RR1	0.363	0.147	0.899
NRAS Q61 (0/1)	Tumor Localization (left/right)	RR2	2.755	2.539	2.971
NRAS Q61 (0/1)	Tumor Localization (left/right)	RR3	1.510	1.042	2.188
NRAS Q61 (0/1)	HP differentiation model 1 (1/2)	OR	1.875	0.646	5.445
NRAS Q61 (0/1)	HP differentiation model 1 (1/2)	RR1	0.774	0.502	1.193
NRAS Q61 (0/1)	HP differentiation model 1 (1/2)	RR2	1.292	1.020	1.564
NRAS Q61 (0/1)	HP differentiation model 1 (1/2)	RR3	1.452	0.761	2.768
NRAS Q61 (0/1)	HP differentiation model 2 (1/2)	OR	0.200	0.022	1.832
NRAS Q61 (0/1)	HP differentiation model 2 (1/2)	RR1	4.355	0.542	35.002
NRAS Q61 (0/1)	HP differentiation model 2 (1/2)	RR2	0.230	-3.583	4.043
NRAS Q61 (0/1)	HP differentiation model 2 (1/2)	RR3	0.871	0.734	1.034
NRAS Q61 (0/1)	For HP phenotype with tubular pattern (0/1)	OR	0.192	0.020	1.832

NRAS Q61 (0/1)	For HP phenotype with tubular pattern (0/1)	RR1	1.136	0.959	1.346
NRAS Q61 (0/1)	For HP phenotype with tubular pattern (0/1)	RR2	0.880	0.703	1.057
NRAS Q61 (0/1)	For HP phenotype with tubular pattern (0/1)	RR3	0.218	0.026	1.832
NRAS Q61 (0/1)	HP phenotype with cribriform pattern (0/1)	OR	0.789	0.277	2.252
NRAS Q61 (0/1)	HP phenotype with cribriform pattern (0/1)	RR1	1.103	0.712	1.710
NRAS Q61 (0/1)	HP phenotype with cribriform pattern (0/1)	RR2	0.907	0.516	1.298
NRAS Q61 (0/1)	HP phenotype with cribriform pattern (0/1)	RR3	0.871	0.472	1.606
NRAS Q61 (0/1)	Mucoid HP phenotype (0/1)	OR	0.263	0.064	1.083
NRAS Q61 (0/1)	Mucoid HP phenotype (0/1)	RR1	2.903	0.890	9.472
NRAS Q61 (0/1)	Mucoid HP phenotype (0/1)	RR2	0.344	-1.669	2.357
NRAS Q61 (0/1)	Mucoid HP phenotype (0/1)	RR3	0.762	0.578	1.005
NRAS Q61 (0/1)	Necrosis (0/1)	OR	2.670	0.886	8.046
NRAS Q61 (0/1)	Necrosis (0/1)	RR1	0.536	0.263	1.094
NRAS Q61 (0/1)	Necrosis (0/1)	RR2	1.866	1.593	2.139
NRAS Q61 (0/1)	Necrosis (0/1)	RR3	1.431	0.941	2.175
NRAS Q61 (0/1)	Limits of invasion model 1 (1/2)	OR	0.417	0.036	4.869
NRAS Q61 (0/1)	Limits of invasion model 1 (1/2)	RR1	1.045	0.923	1.184
NRAS Q61 (0/1)	Limits of invasion model 1 (1/2)	RR2	0.957	0.835	1.079
NRAS Q61 (0/1)	Limits of invasion model 1 (1/2)	RR3	0.435	0.042	4.541
NRAS Q61 (0/1)	Limits of invasion model 2 (1/2)	OR	1.361	0.434	4.270
NRAS Q61 (0/1)	Limits of invasion model 2 (1/2)	RR1	0.915	0.658	1.272
NRAS Q61 (0/1)	Limits of invasion model 2 (1/2)	RR2	1.093	0.836	1.350
NRAS Q61 (0/1)	Limits of invasion model 2 (1/2)	RR3	1.244	0.550	2.815
NRAS Q61 (0/1)	Limits of invasion model 3 (1/2)	OR	0.919	0.320	2.637
NRAS Q61 (0/1)	Limits of invasion model 3 (1/2)	RR1	1.034	0.680	1.574
NRAS Q61 (0/1)	Limits of invasion model 3 (1/2)	RR2	0.967	0.613	1.321
NRAS Q61 (0/1)	Limits of invasion model 3 (1/2)	RR3	0.950	0.504	1.793
NRAS Q61 (0/1)	Limits of invasion model 4 (1/2)	OR	0.909	0.307	2.696

NRAS Q61 (0/1)	Limits of invasion model 4 (1/2)	RR1	1.065	0.521	2.174
NRAS Q61 (0/1)	Limits of invasion model 4 (1/2)	RR2	0.939	0.395	1.483
NRAS Q61 (0/1)	Limits of invasion model 4 (1/2)	RR3	0.968	0.666	1.406
NRAS Q61 (0/1)	Desmoplasic Reaction Model 1	OR	3.333	0.319	34.830
NRAS Q61 (0/1)	Desmoplasic Reaction Model 1	RR1	0.825	0.588	1.158
NRAS Q61 (0/1)	Desmoplasic Reaction Model 1	RR2	1.212	0.975	1.449
NRAS Q61 (0/1)	Desmoplasic Reaction Model 1	RR3	2.750	0.353	21.414
NRAS Q61 (0/1)	Desmoplasic Reaction Model 2	OR	2.917	0.594	14.327
NRAS Q61 (0/1)	Desmoplasic Reaction Model 2	OR	4.800	0.459	50.155
NRAS Q61 (0/1)	Desmoplasic Reaction Model 2	RR1	0.589	0.272	1.278
NRAS Q61 (0/1)	Desmoplasic Reaction Model 2	RR2	1.698	1.381	2.015
NRAS Q61 (0/1)	Desmoplasic Reaction Model 2	RR3	1.719	0.721	4.098
NRAS Q61 (0/1)	Desmoplasic Reaction Model 2	RR3	1.760	0.934	3.317
NRAS Q61 (0/1)	Mixed peri- and/or intratumoral infiltrate Model 1	OR	0.750	0.240	2.341
NRAS Q61 (0/1)	Mixed peri- and/or intratumoral infiltrate Model 1	RR1	1.167	0.629	2.163
NRAS Q61 (0/1)	Mixed peri- and/or intratumoral infiltrate Model 1	RR2	0.857	0.319	1.395
NRAS Q61 (0/1)	Mixed peri- and/or intratumoral infiltrate Model 1	RR3	0.875	0.518	1.477
NRAS Q61 (0/1)	Mixed peri- and/or intratumoral infiltrate Model 2	OR	0.844	0.246	2.904
NRAS Q61 (0/1)	Mixed peri- and/or intratumoral infiltrate Model 2	RR1	1.125	0.474	2.670
NRAS Q61 (0/1)	Mixed peri- and/or intratumoral infiltrate Model 2	RR2	0.889	0.238	1.540
NRAS Q61 (0/1)	Mixed peri- and/or intratumoral infiltrate Model 2	RR3	0.950	0.655	1.378
NRAS Q61 (0/1)	Mixed peri- and/or intratumoral infiltrate Model 3	OR	1.082	0.252	4.645
NRAS Q61 (0/1)	Mixed peri- and/or intratumoral infiltrate Model 3	RR1	0.938	0.286	3.072
NRAS Q61 (0/1)	Mixed peri- and/or intratumoral infiltrate Model 3	RR2	1.066	0.414	1.718
NRAS Q61 (0/1)	Mixed peri- and/or intratumoral infiltrate Model 3	RR3	1.015	0.775	1.329
NRAS Q61 (0/1)	Lymphocyte peri- and/or intratumoral Model 1	OR	1.167	0.379	3.587
NRAS Q61 (0/1)	Lymphocyte peri- and/or intratumoral Model 1	RR1	0.933	0.567	1.538
NRAS Q61 (0/1)	Lymphocyte peri- and/or intratumoral Model 1	RR2	1.072	0.706	1.438

NRAS Q61 (0/1)	Lymphocyte peri- and/or intratumoral Model 1	RR3	1.089	0.583	2.034
NRAS Q61 (0/1)	Lymphocyte peri- and/or intratumoral Model 2	OR	0.458	0.106	1.985
NRAS Q61 (0/1)	Lymphocyte peri- and/or intratumoral Model 2	RR1	1.867	0.560	6.223
NRAS Q61 (0/1)	Lymphocyte peri- and/or intratumoral Model 2	RR2	0.536	-0.771	1.843
NRAS Q61 (0/1)	Lymphocyte peri- and/or intratumoral Model 2	RR3	0.856	0.648	1.129
NRAS Q61 (0/1)	Lymphocyte peri- and/or intratumoral Model 3	OR	1.450	0.086	24.565
NRAS Q61 (0/1)	Lymphocyte peri- and/or intratumoral Model 3	RR1	0.700	0.046	10.575
NRAS Q61 (0/1)	Lymphocyte peri- and/or intratumoral Model 3	RR2	1.429	0.775	2.083
NRAS Q61 (0/1)	Lymphocyte peri- and/or intratumoral Model 3	RR3	1.015	0.903	1.140
NRAS Q61 (0/1)	PMN peri- and/or intratumoral infiltrate Model 1	OR	0.567	0.171	1.883
NRAS Q61 (0/1)	PMN peri- and/or intratumoral infiltrate Model 1	RR1	1.448	0.649	3.233
NRAS Q61 (0/1)	PMN peri- and/or intratumoral infiltrate Model 1	RR2	0.691	-0.108	1.490
NRAS Q61 (0/1)	PMN peri- and/or intratumoral infiltrate Model 1	RR3	0.821	0.546	1.234
NRAS Q61 (0/1)	PMN peri- and/or intratumoral infiltrate Model 2	OR	0.370	0.087	1.585
NRAS Q61 (0/1)	PMN peri- and/or intratumoral infiltrate Model 2	RR1	2.172	0.668	7.068
NRAS Q61 (0/1)	PMN peri- and/or intratumoral infiltrate Model 2	RR2	0.460	-1.044	1.964
NRAS Q61 (0/1)	PMN peri- and/or intratumoral infiltrate Model 2	RR3	0.805	0.596	1.086
NRAS Q61 (0/1)	PMN peri- and/or intratumoral infiltrate Model 3	OR	0.912	0.139	6.005
NRAS Q61 (0/1)	PMN peri- and/or intratumoral infiltrate Model 3	RR1	1.086	0.199	5.939
NRAS Q61 (0/1)	PMN peri- and/or intratumoral infiltrate Model 3	RR2	0.921	0.034	1.808
NRAS Q61 (0/1)	PMN peri- and/or intratumoral infiltrate Model 3	RR3	0.991	0.823	1.193
NRAS G12/G13 (0/1)	Tumor Localization (left/right)	OR	3.482	0.692	17.515
NRAS G12/G13 (0/1)	Tumor Localization (left/right)	RR1	0.382	0.099	1.479
NRAS G12/G13 (0/1)	Tumor Localization (left/right)	RR2	2.618	2.335	2.901
NRAS G12/G13 (0/1)	Tumor Localization (left/right)	RR3	1.331	0.991	1.788
NRAS G12/G13 (0/1)	HP differentiation model 1 (1/2)	OR	1.338	0.409	4.374
NRAS G12/G13 (0/1)	HP differentiation model 1 (1/2)	RR1	0.882	0.518	1.501
NRAS G12/G13 (0/1)	HP differentiation model 1 (1/2)	RR2	1.134	0.770	1.498

NRAS G12/G13 (0/1)	HP differentiation model 1 (1/2)	RR3	1.180	0.613	2.273
NRAS G12/G13 (0/1)	HP differentiation model 2 (1/2)	OR	0.300	0.053	1.684
NRAS G12/G13 (0/1)	HP differentiation model 2 (1/2)	RR1	2.867	0.647	12.700
NRAS G12/G13 (0/1)	HP differentiation model 2 (1/2)	RR2	0.349	-1.871	2.569
NRAS G12/G13 (0/1)	HP differentiation model 2 (1/2)	RR3	0.860	0.659	1.122
NRAS G12/G13 (0/1)	HP phenotype with cribriform pattern (0/1)	OR	1.338	0.409	4.374
NRAS G12/G13 (0/1)	HP phenotype with cribriform pattern (0/1)	RR1	0.882	0.518	1.501
NRAS G12/G13 (0/1)	HP phenotype with cribriform pattern (0/1)	RR2	1.134	0.770	1.498
NRAS G12/G13 (0/1)	HP phenotype with cribriform pattern (0/1)	RR3	1.180	0.613	2.273
NRAS G12/G13 (0/1)	Mucoid HP phenotype (0/1)	OR	0.728	0.187	2.836
NRAS G12/G13 (0/1)	Mucoid HP phenotype (0/1)	RR1	1.274	0.459	3.536
NRAS G12/G13 (0/1)	Mucoid HP phenotype (0/1)	RR2	0.785	-0.030	1.600
NRAS G12/G13 (0/1)	Mucoid HP phenotype (0/1)	RR3	0.927	0.659	1.305
NRAS G12/G13 (0/1)	Necrosis (0/1)	OR	1.798	0.491	6.581
NRAS G12/G13 (0/1)	Necrosis (0/1)	RR1	0.675	0.270	1.687
NRAS G12/G13 (0/1)	Necrosis (0/1)	RR2	1.481	1.076	1.886
NRAS G12/G13 (0/1)	Necrosis (0/1)	RR3	1.213	0.822	1.790
NRAS G12/G13 (0/1)	Limits of invasion model 1 (1/2)	RR1	1.075	0.991	1.167
NRAS G12/G13 (0/1)	Limits of invasion model 1 (1/2)	RR2	0.930	0.846	1.014
NRAS G12/G13 (0/1)	Limits of invasion model 2 (1/2)	OR	0.839	0.225	3.130
NRAS G12/G13 (0/1)	Limits of invasion model 2 (1/2)	RR1	1.051	0.731	1.511
NRAS G12/G13 (0/1)	Limits of invasion model 2 (1/2)	RR2	0.951	0.631	1.271
NRAS G12/G13 (0/1)	Limits of invasion model 2 (1/2)	RR3	0.882	0.340	2.290
NRAS G12/G13 (0/1)	Limits of invasion model 3 (1/2)	OR	1.020	0.307	3.386
NRAS G12/G13 (0/1)	Limits of invasion model 3 (1/2)	RR1	0.992	0.615	1.602
NRAS G12/G13 (0/1)	Limits of invasion model 3 (1/2)	RR2	1.008	0.631	1.385
NRAS G12/G13 (0/1)	Limits of invasion model 3 (1/2)	RR3	1.012	0.492	2.082
NRAS G12/G13 (0/1)	Limits of invasion model 4 (1/2)	OR	1.071	0.309	3.715

NRAS G12/G13 (0/1)	Limits of invasion model 4 (1/2)	RR1	0.956	0.419	2.178
NRAS G12/G13 (0/1)	Limits of invasion model 4 (1/2)	RR2	1.046	0.509	1.583
NRAS G12/G13 (0/1)	Limits of invasion model 4 (1/2)	RR3	1.024	0.673	1.557
NRAS G12/G13 (0/1)	Desmoplasic Reaction Model 1	RR1	1.313	1.033	1.667
NRAS G12/G13 (0/1)	Desmoplasic Reaction Model 1	RR2	0.762	0.482	1.042
NRAS G12/G13 (0/1)	Desmoplasic Reaction Model 2	OR	2.200	0.329	14.726
NRAS G12/G13 (0/1)	Desmoplasic Reaction Model 2	RR1	0.636	0.191	2.119
NRAS G12/G13 (0/1)	Desmoplasic Reaction Model 2	RR2	1.572	1.127	2.017
NRAS G12/G13 (0/1)	Desmoplasic Reaction Model 2	RR3	1.400	0.680	2.882
NRAS G12/G13 (0/1)	Mixed peri- and/or intratumoral infiltrate Model 1	OR	0.544	0.146	2.035
NRAS G12/G13 (0/1)	Mixed peri- and/or intratumoral infiltrate Model 1	RR1	1.349	0.737	2.468
NRAS G12/G13 (0/1)	Mixed peri- and/or intratumoral infiltrate Model 1	RR2	0.741	0.129	1.353
NRAS G12/G13 (0/1)	Mixed peri- and/or intratumoral infiltrate Model 1	RR3	0.734	0.355	1.517
NRAS G12/G13 (0/1)	Mixed peri- and/or intratumoral infiltrate Model 2	OR	0.321	0.083	1.250
NRAS G12/G13 (0/1)	Mixed peri- and/or intratumoral infiltrate Model 2	RR1	2.056	0.922	4.584
NRAS G12/G13 (0/1)	Mixed peri- and/or intratumoral infiltrate Model 2	RR2	0.486	-0.648	1.620
NRAS G12/G13 (0/1)	Mixed peri- and/or intratumoral infiltrate Model 2	RR3	0.661	0.365	1.197
NRAS G12/G13 (0/1)	Mixed peri- and/or intratumoral infiltrate Model 3	OR	0.581	0.121	2.797
NRAS G12/G13 (0/1)	Mixed peri- and/or intratumoral infiltrate Model 3	RR1	1.542	0.454	5.240
NRAS G12/G13 (0/1)	Mixed peri- and/or intratumoral infiltrate Model 3	RR2	0.649	-0.439	1.737
NRAS G12/G13 (0/1)	Mixed peri- and/or intratumoral infiltrate Model 3	RR3	0.895	0.627	1.278
NRAS G12/G13 (0/1)	Lymphocyte peri- and/or intratumoral Model 1	OR	0.833	0.225	3.085
NRAS G12/G13 (0/1)	Lymphocyte peri- and/or intratumoral Model 1	RR1	1.083	0.619	1.896
NRAS G12/G13 (0/1)	Lymphocyte peri- and/or intratumoral Model 1	RR2	0.923	0.459	1.387
NRAS G12/G13 (0/1)	Lymphocyte peri- and/or intratumoral Model 1	RR3	0.903	0.426	1.912
NRAS G12/G13 (0/1)	Lymphocyte peri- and/or intratumoral Model 2	OR	0.774	0.169	3.541
NRAS G12/G13 (0/1)	Lymphocyte peri- and/or intratumoral Model 2	RR1	1.219	0.383	3.882
NRAS G12/G13 (0/1)	Lymphocyte peri- and/or intratumoral Model 2	RR2	0.820	-0.016	1.656

NRAS G12/G13 (0/1)	Lymphocyte peri- and/or intratumoral Model 2	RR3	0.944	0.656	1.357
NRAS G12/G13 (0/1)	Lymphocyte peri- and/or intratumoral Model 3	RR3	1.054	0.980	1.134
NRAS G12/G13 (0/1)	PMN peri- and/or intratumoral infiltrate Model 1	OR	1.167	0.297	4.588
NRAS G12/G13 (0/1)	PMN peri- and/or intratumoral infiltrate Model 1	RR1	0.905	0.367	2.230
NRAS G12/G13 (0/1)	PMN peri- and/or intratumoral infiltrate Model 1	RR2	1.105	0.567	1.643
NRAS G12/G13 (0/1)	PMN peri- and/or intratumoral infiltrate Model 1	RR3	1.056	0.661	1.686
NRAS G12/G13 (0/1)	PMN peri- and/or intratumoral infiltrate Model 2	OR	1.786	0.332	9.592
NRAS G12/G13 (0/1)	PMN peri- and/or intratumoral infiltrate Model 2	RR1	0.633	0.161	2.499
NRAS G12/G13 (0/1)	PMN peri- and/or intratumoral infiltrate Model 2	RR2	1.580	1.108	2.052
NRAS G12/G13 (0/1)	PMN peri- and/or intratumoral infiltrate Model 2	RR3	1.131	0.824	1.552
NRAS G12/G13 (0/1)	PMN peri- and/or intratumoral infiltrate Model 3	OR	1.294	0.130	12.835
NRAS G12/G13 (0/1)	PMN peri- and/or intratumoral infiltrate Model 3	RR1	0.792	0.098	6.420
NRAS G12/G13 (0/1)	PMN peri- and/or intratumoral infiltrate Model 3	RR2	1.263	0.569	1.957
NRAS G12/G13 (0/1)	PMN peri- and/or intratumoral infiltrate Model 3	RR3	1.025	0.837	1.254
BRAF (0/1)	Tumor Localization (left/right)	OR	1.939	0.562	6.698
BRAF (0/1)	Tumor Localization (left/right)	RR1	0.640	0.287	1.427
BRAF (0/1)	Tumor Localization (left/right)	RR2	1.563	1.210	1.916
BRAF (0/1)	Tumor Localization (left/right)	RR3	1.240	0.792	1.943
BRAF (0/1)	HP differentiation model 1 (1/2)	OR	1.080	0.326	3.577
BRAF (0/1)	HP differentiation model 1 (1/2)	RR1	0.969	0.597	1.574
BRAF (0/1)	HP differentiation model 1 (1/2)	RR2	1.032	0.660	1.404
BRAF (0/1)	HP differentiation model 1 (1/2)	RR3	1.047	0.513	2.135
BRAF (0/1)	HP differentiation model 2 (1/2)	OR	0.543	0.058	5.062
BRAF (0/1)	HP differentiation model 2 (1/2)	RR1	1.744	0.221	13.754
BRAF (0/1)	HP differentiation model 2 (1/2)	RR2	0.573	-0.950	2.096
BRAF (0/1)	HP differentiation model 2 (1/2)	RR3	0.947	0.796	1.126
BRAF (0/1)	For HP phenotype with tubular pattern (0/1)	OR	1.436	0.148	13.965
BRAF (0/1)	For HP phenotype with tubular pattern (0/1)	RR1	0.972	0.823	1.147

BRAF (0/1)	For HP phenotype with tubular pattern (0/1)	RR2	1.029	0.880	1.178
BRAF (0/1)	For HP phenotype with tubular pattern (0/1)	RR3	1.395	0.169	11.521
BRAF (0/1)	HP phenotype with cribriform pattern (0/1)	OR	0.747	0.229	2.442
BRAF (0/1)	HP phenotype with cribriform pattern (0/1)	RR1	1.134	0.666	1.929
BRAF (0/1)	HP phenotype with cribriform pattern (0/1)	RR2	0.882	0.414	1.350
BRAF (0/1)	HP phenotype with cribriform pattern (0/1)	RR3	0.847	0.440	1.631
BRAF (0/1)	Mucoid HP phenotype (0/1)	OR	1.374	0.353	5.352
BRAF (0/1)	Mucoid HP phenotype (0/1)	RR1	0.785	0.283	2.178
BRAF (0/1)	Mucoid HP phenotype (0/1)	RR2	1.274	0.772	1.776
BRAF (0/1)	Mucoid HP phenotype (0/1)	RR3	1.078	0.766	1.517
BRAF (0/1)	Necrosis (0/1)	OR	1.244	0.372	4.167
BRAF (0/1)	Necrosis (0/1)	RR1	0.872	0.415	1.832
BRAF (0/1)	Necrosis (0/1)	RR2	1.147	0.690	1.604
BRAF (0/1)	Necrosis (0/1)	RR3	1.085	0.680	1.732
BRAF (0/1)	Limits of invasion model 1 (1/2)	OR	0.155	0.013	1.848
BRAF (0/1)	Limits of invasion model 1 (1/2)	RR1	1.127	0.919	1.382
BRAF (0/1)	Limits of invasion model 1 (1/2)	RR2	0.887	0.679	1.095
BRAF (0/1)	Limits of invasion model 1 (1/2)	RR3	0.174	0.017	1.788
BRAF (0/1)	Limits of invasion model 2 (1/2)	OR	1.192	0.319	4.445
BRAF (0/1)	Limits of invasion model 2 (1/2)	RR1	0.951	0.662	1.368
BRAF (0/1)	Limits of invasion model 2 (1/2)	RR2	1.052	0.763	1.341
BRAF (0/1)	Limits of invasion model 2 (1/2)	RR3	1.134	0.437	2.944
BRAF (0/1)	Limits of invasion model 3 (1/2)	OR	0.981	0.295	3.257
BRAF (0/1)	Limits of invasion model 3 (1/2)	RR1	1.008	0.624	1.626
BRAF (0/1)	Limits of invasion model 3 (1/2)	RR2	0.992	0.608	1.376
BRAF (0/1)	Limits of invasion model 3 (1/2)	RR3	0.988	0.480	2.034
BRAF (0/1)	Limits of invasion model 4 (1/2)	OR	0.933	0.269	3.236
BRAF (0/1)	Limits of invasion model 4 (1/2)	RR1	1.047	0.459	2.386

BRAF (0/1)	Limits of invasion model 4 (1/2)	RR2	0.955	0.367	1.543
BRAF (0/1)	Limits of invasion model 4 (1/2)	RR3	0.977	0.642	1.486
BRAF (0/1)	Desmoplasic Reaction Model 1	OR	1.500	0.138	16.268
BRAF (0/1)	Desmoplasic Reaction Model 1	RR1	0.933	0.642	1.356
BRAF (0/1)	Desmoplasic Reaction Model 1	RR2	1.072	0.781	1.363
BRAF (0/1)	Desmoplasic Reaction Model 1	RR3	1.400	0.187	10.503
BRAF (0/1)	Desmoplasic Reaction Model 2	OR	1.630	0.287	9.256
BRAF (0/1)	Desmoplasic Reaction Model 2	RR1	0.788	0.352	1.760
BRAF (0/1)	Desmoplasic Reaction Model 2	RR2	1.269	0.833	1.705
BRAF (0/1)	Desmoplasic Reaction Model 2	RR3	1.283	0.500	3.294
BRAF (0/1)	Mixed peri- and/or intratumoral infiltrate Model 1	OR	0.754	0.202	2.812
BRAF (0/1)	Mixed peri- and/or intratumoral infiltrate Model 1	RR1	1.168	0.553	2.464
BRAF (0/1)	Mixed peri- and/or intratumoral infiltrate Model 1	RR2	0.856	0.241	1.471
BRAF (0/1)	Mixed peri- and/or intratumoral infiltrate Model 1	RR3	0.880	0.497	1.560
BRAF (0/1)	Mixed peri- and/or intratumoral infiltrate Model 2	OR	1.182	0.294	4.754
BRAF (0/1)	Mixed peri- and/or intratumoral infiltrate Model 2	RR1	0.892	0.348	2.286
BRAF (0/1)	Mixed peri- and/or intratumoral infiltrate Model 2	RR2	1.121	0.577	1.665
BRAF (0/1)	Mixed peri- and/or intratumoral infiltrate Model 2	RR3	1.054	0.671	1.656
BRAF (0/1)	Mixed peri- and/or intratumoral infiltrate Model 3	OR	1.722	0.358	8.295
BRAF (0/1)	Mixed peri- and/or intratumoral infiltrate Model 3	RR1	0.649	0.191	2.205
BRAF (0/1)	Mixed peri- and/or intratumoral infiltrate Model 3	RR2	1.541	1.083	1.999
BRAF (0/1)	Mixed peri- and/or intratumoral infiltrate Model 3	RR3	1.117	0.782	1.595
BRAF (0/1)	Lymphocyte peri- and/or intratumoral Model 1	OR	1.440	0.398	5.211
BRAF (0/1)	Lymphocyte peri- and/or intratumoral Model 1	RR1	0.855	0.506	1.446
BRAF (0/1)	Lymphocyte peri- and/or intratumoral Model 1	RR2	1.170	0.821	1.519
BRAF (0/1)	Lymphocyte peri- and/or intratumoral Model 1	RR3	1.232	0.573	2.646
BRAF (0/1)	Lymphocyte peri- and/or intratumoral Model 2	OR	1.125	0.249	5.080
BRAF (0/1)	Lymphocyte peri- and/or intratumoral Model 2	RR1	0.912	0.284	2.933

BRAF (0/1)	Lymphocyte peri- and/or intratumoral Model 2	RR2	1.096	0.468	1.724
BRAF (0/1)	Lymphocyte peri- and/or intratumoral Model 2	RR3	1.026	0.730	1.442
BRAF (0/1)	Lymphocyte peri- and/or intratumoral Model 3	OR	3.083	0.179	53.158
BRAF (0/1)	Lymphocyte peri- and/or intratumoral Model 3	RR1	0.342	0.023	5.087
BRAF (0/1)	Lymphocyte peri- and/or intratumoral Model 3	RR2	2.924	2.605	3.243
BRAF (0/1)	Lymphocyte peri- and/or intratumoral Model 3	RR3	1.055	0.894	1.245
BRAF (0/1)	PMN peri- and/or intratumoral infiltrate Model 1	OR	0.857	0.218	3.371
BRAF (0/1)	PMN peri- and/or intratumoral infiltrate Model 1	RR1	1.105	0.448	2.724
BRAF (0/1)	PMN peri- and/or intratumoral infiltrate Model 1	RR2	0.905	0.248	1.562
BRAF (0/1)	PMN peri- and/or intratumoral infiltrate Model 1	RR3	0.947	0.593	1.513
BRAF (0/1)	PMN peri- and/or intratumoral infiltrate Model 2	OR	1.074	0.238	4.840
BRAF (0/1)	PMN peri- and/or intratumoral infiltrate Model 2	RR1	0.947	0.305	2.945
BRAF (0/1)	PMN peri- and/or intratumoral infiltrate Model 2	RR2	1.056	0.414	1.698
BRAF (0/1)	PMN peri- and/or intratumoral infiltrate Model 2	RR3	1.018	0.702	1.475
BRAF (0/1)	PMN peri- and/or intratumoral infiltrate Model 3	OR	0.773	0.078	7.664
BRAF (0/1)	PMN peri- and/or intratumoral infiltrate Model 3	RR1	1.263	0.156	10.243
BRAF (0/1)	PMN peri- and/or intratumoral infiltrate Model 3	RR2	0.792	-0.315	1.899
BRAF (0/1)	PMN peri- and/or intratumoral infiltrate Model 3	RR3	0.976	0.797	1.195
EGFR (0/1)	Tumor Localization (left/right)	RR3	0.702	0.592	0.831
EGFR (0/1)	HP differentiation model 1 (1/2)	RR1	0.579	0.464	0.722
EGFR (0/1)	HP differentiation model 1 (1/2)	RR2	1.727	1.612	1.842
EGFR (0/1)	HP differentiation model 2 (1/2)	RR3	0.895	0.819	0.978
EGFR (0/1)	For HP phenotype with tubular pattern (0/1)	RR1	0.912	0.842	0.989
EGFR (0/1)	For HP phenotype with tubular pattern (0/1)	RR2	1.096	1.026	1.166
EGFR (0/1)	HP phenotype with cribriform pattern (0/1)	RR1	0.579	0.464	0.722
EGFR (0/1)	HP phenotype with cribriform pattern (0/1)	RR2	1.727	1.612	1.842
EGFR (0/1)	Mucoid HP phenotype (0/1)	RR1	0.211	0.127	0.348
EGFR (0/1)	Mucoid HP phenotype (0/1)	RR2	4.739	4.655	4.823

EGFR (0/1)	Necrosis (0/1)	RR3	0.632	0.518	0.770
EGFR (0/1)	Limits of invasion model 1 (1/2)	RR1	0.947	0.891	1.007
EGFR (0/1)	Limits of invasion model 1 (1/2)	RR2	1.056	1.000	1.112
EGFR (0/1)	Limits of invasion model 2 (1/2)	RR1	0.702	0.592	0.831
EGFR (0/1)	Limits of invasion model 2 (1/2)	RR2	1.425	1.315	1.535
EGFR (0/1)	Limits of invasion model 3 (1/2)	RR1	0.596	0.482	0.738
EGFR (0/1)	Limits of invasion model 3 (1/2)	RR2	1.678	1.564	1.792
EGFR (0/1)	Limits of invasion model 4 (1/2)	RR1	0.333	0.231	0.481
EGFR (0/1)	Limits of invasion model 4 (1/2)	RR2	3.003	2.901	3.105
EGFR (0/1)	Desmoplasic Reaction Model 1	RR3	0.154	0.062	0.379
EGFR (0/1)	Desmoplasic Reaction Model 2	RR3	0.500	0.340	0.734
EGFR (0/1)	Lymphocyte peri- and/or intratumoral Model 1	RR3	0.440	0.322	0.602
EGFR (0/1)	Lymphocyte peri- and/or intratumoral Model 2	RR3	0.780	0.673	0.904
EGFR (0/1)	Lymphocyte peri- and/or intratumoral Model 3	RR3	0.960	0.907	1.016
EGFR (0/1)	PMN peri- and/or intratumoral infiltrate Model 1	RR1	0.347	0.236	0.509
EGFR (0/1)	PMN peri- and/or intratumoral infiltrate Model 1	RR2	2.882	2.771	2.993
EGFR (0/1)	PMN peri- and/or intratumoral infiltrate Model 2	RR1	0.224	0.133	0.378
EGFR (0/1)	PMN peri- and/or intratumoral infiltrate Model 2	RR2	4.464	4.373	4.555
EGFR (0/1)	PMN peri- and/or intratumoral infiltrate Model 3	RR1	0.082	0.032	0.209
EGFR (0/1)	PMN peri- and/or intratumoral infiltrate Model 3	RR2	12.195	12.145	12.245
Any NRAS-type (0/1)	Tumor Localization (left/right)	RR3	1.515	1.242	1.849
Any NRAS-type (0/1)	HP differentiation model 1 (1/2)	OR	1.500	0.336	6.702
Any NRAS-type (0/1)	HP differentiation model 1 (1/2)	RR1	0.833	0.402	1.727
Any NRAS-type (0/1)	HP differentiation model 1 (1/2)	RR2	1.200	0.769	1.631
Any NRAS-type (0/1)	HP differentiation model 1 (1/2)	RR3	1.250	0.578	2.704
Any NRAS-type (0/1)	HP differentiation model 2 (1/2)	OR	0.106	0.017	0.674
Any NRAS-type (0/1)	HP differentiation model 2 (1/2)	RR1	6.250	1.517	25.744
Any NRAS-type (0/1)	HP differentiation model 2 (1/2)	RR2	0.160	-4.573	4.893

Any NRAS-type (0/1)	HP differentiation model 2 (1/2)	RR3	0.665	0.387	1.142
Any NRAS-type (0/1)	For HP phenotype with tubular pattern (0/1)	RR3	1.111	1.013	1.219
Any NRAS-type (0/1)	HP phenotype with cribriform pattern (0/1)	OR	0.829	0.178	3.856
Any NRAS-type (0/1)	HP phenotype with cribriform pattern (0/1)	RR1	1.078	0.600	1.937
Any NRAS-type (0/1)	HP phenotype with cribriform pattern (0/1)	RR2	0.928	0.450	1.406
Any NRAS-type (0/1)	HP phenotype with cribriform pattern (0/1)	RR3	0.893	0.345	2.313
Any NRAS-type (0/1)	Mucoid HP phenotype (0/1)	OR	0.417	0.085	2.044
Any NRAS-type (0/1)	Mucoid HP phenotype (0/1)	RR1	1.875	0.655	5.371
Any NRAS-type (0/1)	Mucoid HP phenotype (0/1)	RR2	0.533	-0.687	1.753
Any NRAS-type (0/1)	Mucoid HP phenotype (0/1)	RR3	0.781	0.449	1.360
Any NRAS-type (0/1)	Necrosis (0/1)	OR	4.667	0.533	40.886
Any NRAS-type (0/1)	Necrosis (0/1)	RR1	0.313	0.048	2.017
Any NRAS-type (0/1)	Necrosis (0/1)	RR2	3.195	2.930	3.460
Any NRAS-type (0/1)	Necrosis (0/1)	RR3	1.458	1.032	2.062
Any NRAS-type (0/1)	Limits of invasion model 1 (1/2)	RR1	1.064	0.992	1.141
Any NRAS-type (0/1)	Limits of invasion model 1 (1/2)	RR2	0.940	0.868	1.012
Any NRAS-type (0/1)	Limits of invasion model 2 (1/2)	OR	1.543	0.325	7.333
Any NRAS-type (0/1)	Limits of invasion model 2 (1/2)	RR1	0.868	0.494	1.526
Any NRAS-type (0/1)	Limits of invasion model 2 (1/2)	RR2	1.152	0.778	1.526
Any NRAS-type (0/1)	Limits of invasion model 2 (1/2)	RR3	1.339	0.493	3.637
Any NRAS-type (0/1)	Limits of invasion model 3 (1/2)	OR	1.632	0.364	7.305
Any NRAS-type (0/1)	Limits of invasion model 3 (1/2)	RR1	0.806	0.390	1.667
Any NRAS-type (0/1)	Limits of invasion model 3 (1/2)	RR2	1.241	0.825	1.657
Any NRAS-type (0/1)	Limits of invasion model 3 (1/2)	RR3	1.316	0.604	2.865
Any NRAS-type (0/1)	Limits of invasion model 4 (1/2)	OR	1.688	0.308	9.249
Any NRAS-type (0/1)	Limits of invasion model 4 (1/2)	RR1	0.694	0.198	2.438
Any NRAS-type (0/1)	Limits of invasion model 4 (1/2)	RR2	1.441	0.945	1.937
Any NRAS-type (0/1)	Limits of invasion model 4 (1/2)	RR3	1.172	0.747	1.839

Any NRAS-type (0/1)	Desmoplasic Reaction Model 1	RR1	1.263	1.029	1.551
Any NRAS-type (0/1)	Desmoplasic Reaction Model 1	RR2	0.792	0.558	1.026
Any NRAS-type (0/1)	Desmoplasic Reaction Model 2	OR	2.000	0.159	25.115
Any NRAS-type (0/1)	Desmoplasic Reaction Model 2	RR1	0.667	0.128	3.470
Any NRAS-type (0/1)	Desmoplasic Reaction Model 2	RR2	1.499	0.960	2.038
Any NRAS-type (0/1)	Desmoplasic Reaction Model 2	RR3	1.333	0.545	3.262
Any NRAS-type (0/1)	Mixed peri- and/or intratumoral infiltrate Model 1	OR	0.870	0.157	4.802
Any NRAS-type (0/1)	Mixed peri- and/or intratumoral infiltrate Model 1	RR1	1.075	0.454	2.545
Any NRAS-type (0/1)	Mixed peri- and/or intratumoral infiltrate Model 1	RR2	0.930	0.309	1.551
Any NRAS-type (0/1)	Mixed peri- and/or intratumoral infiltrate Model 1	RR3	0.935	0.401	2.181
Any NRAS-type (0/1)	Mixed peri- and/or intratumoral infiltrate Model 2	OR	0.387	0.068	2.191
Any NRAS-type (0/1)	Mixed peri- and/or intratumoral infiltrate Model 2	RR1	1.792	0.705	4.556
Any NRAS-type (0/1)	Mixed peri- and/or intratumoral infiltrate Model 2	RR2	0.558	-0.529	1.645
Any NRAS-type (0/1)	Mixed peri- and/or intratumoral infiltrate Model 2	RR3	0.694	0.305	1.577
Any NRAS-type (0/1)	Mixed peri- and/or intratumoral infiltrate Model 3	OR	1.143	0.117	11.177
Any NRAS-type (0/1)	Mixed peri- and/or intratumoral infiltrate Model 3	RR1	0.896	0.135	5.961
Any NRAS-type (0/1)	Mixed peri- and/or intratumoral infiltrate Model 3	RR2	1.116	0.355	1.877
Any NRAS-type (0/1)	Mixed peri- and/or intratumoral infiltrate Model 3	RR3	1.024	0.696	1.505
Any NRAS-type (0/1)	Lymphocyte peri- and/or intratumoral Model 1	OR	0.900	0.180	4.504
Any NRAS-type (0/1)	Lymphocyte peri- and/or intratumoral Model 1	RR1	1.048	0.522	2.101
Any NRAS-type (0/1)	Lymphocyte peri- and/or intratumoral Model 1	RR2	0.954	0.428	1.480
Any NRAS-type (0/1)	Lymphocyte peri- and/or intratumoral Model 1	RR3	0.943	0.378	2.353
Any NRAS-type (0/1)	Lymphocyte peri- and/or intratumoral Model 2	OR	0.643	0.107	3.874
Any NRAS-type (0/1)	Lymphocyte peri- and/or intratumoral Model 2	RR1	1.397	0.378	5.168
Any NRAS-type (0/1)	Lymphocyte peri- and/or intratumoral Model 2	RR2	0.716	-0.303	1.735
Any NRAS-type (0/1)	Lymphocyte peri- and/or intratumoral Model 2	RR3	0.898	0.549	1.469
Any NRAS-type (0/1)	Lymphocyte peri- and/or intratumoral Model 3	RR3	1.048	0.982	1.117
Any NRAS-type (0/1)	PMN peri- and/or intratumoral infiltrate Model 1	OR	0.517	0.093	2.881

Any NRAS-type (0/1)	PMN peri- and/or intratumoral infiltrate Model 1	RR1	1.467	0.597	3.605
Any NRAS-type (0/1)	PMN peri- and/or intratumoral infiltrate Model 1	RR2	0.682	-0.188	1.552
Any NRAS-type (0/1)	PMN peri- and/or intratumoral infiltrate Model 1	RR3	0.759	0.331	1.736
Any NRAS-type (0/1)	PMN peri- and/or intratumoral infiltrate Model 2	OR	0.588	0.094	3.696
Any NRAS-type (0/1)	PMN peri- and/or intratumoral infiltrate Model 2	RR1	1.467	0.418	5.150
Any NRAS-type (0/1)	PMN peri- and/or intratumoral infiltrate Model 2	RR2	0.682	-0.367	1.731
Any NRAS-type (0/1)	PMN peri- and/or intratumoral infiltrate Model 2	RR3	0.863	0.479	1.553
Any NRAS-type (0/1)	PMN peri- and/or intratumoral infiltrate Model 3	OR	0.500	0.046	5.404
Any NRAS-type (0/1)	PMN peri- and/or intratumoral infiltrate Model 3	RR1	1.833	0.244	13.799
Any NRAS-type (0/1)	PMN peri- and/or intratumoral infiltrate Model 3	RR2	0.546	-1.043	2.135
Any NRAS-type (0/1)	PMN peri- and/or intratumoral infiltrate Model 3	RR3	0.917	0.633	1.327
